# Expanding Catalytic Active Surface via Dual-Phase Interfaces for High-Energy-Density Quasi-Solid-State Na–S Batteries

**DOI:** 10.1021/acsnano.6c12130

**Published:** 2026-09-02

**Authors:** Xuan Lu, Sicong Jiang, Hongyang Zhao, Xiaobo Zheng, Yiwen Zhang, Kesong Yang, Shujiang Ding, Xiaofei Hu, Weiyang Li

**Affiliations:** † School of Chemistry, Engineering Research Center of Energy Storage Materials and Devices (Ministry of Education), Xi’an Key Laboratory of Sustainable Energy Materials Chemistry, 12480 Xi’an Jiaotong University, Xi’an, Shaanxi 710049, People’s Republic of China; ‡ Thayer School of Engineering, 3728 Dartmouth College, 14 Engineering Drive, Hanover, New Hampshire 03755, United States; § Aiiso Yufeng Li Family Department of Chemical and NanoEngineering, 8784 University of California San Diego, 9500 Gilman Drive, La Jolla, California 92093-0448, United States; ∥ Centre for Clean Energy Technology, School of Mathematical and Physical Sciences, Faculty of Science, 614632 University of Technology Sydney, Sydney, New South Wales 2007, Australia

**Keywords:** quasi-solid-state sodium−sulfur battery, electrocatalyst, ionic-electronic dual conductor, dual-phase interface, pouch cell

## Abstract

Quasi-solid-state sodium–sulfur (Na–S) batteries are widely regarded as promising candidates for next-generation energy storage due to their higher energy density and enhanced safety over traditional liquid-electrolyte counterparts. However, electrolyte solidification restricts ion transport within the sulfur cathode, leading to poor sulfur utilization and exacerbating sluggish sodium polysulfide (NaPS) conversion kinetics. Current cathode designs mitigate this challenge by introducing a high proportion of conductive additives to expand the sulfur–carbon-electrolyte triple-phase interfaces (TPIs), which inevitably sacrifices the practical energy density of the battery. This challenge becomes more severe under practical conditions of high sulfur contents (50 wt %), where both ionic and electronic pathways are simultaneously obstructed. Here, we develop a presodiated TiO_2_ (Na_*x*_TiO_2_) electrocatalyst possessing ion-electron dual-conductive characteristics via an electrochemical ion-intercalation approach. The introduction of Na_*x*_TiO_2_ extends the active surface beyond conventional TPIs, enabling rapid catalytic conversion of NaPSs directly at the sulfur-Na_*x*_TiO_2_ dual-phase interfaces (DPIs). Mechanistic studies show that Na intercalation modulates the local Ti–O coordination environment and the Ti 3d-O 2p electronic structure, which significantly improves the electronic conductivity and interfacial Na^+^ transfer kinetics of TiO_2_ while reducing the energy barrier for the rate-determining Na_2_S_2_-to-Na_2_S step in NaPS conversion. Consequently, an ultrahigh sulfur utilization exceeding 80% is achieved in a high-sulfur-content cathode (70 wt %), delivering a high initial specific capacity of 1391.2 mAh g^–1^ alongside robust cyclability over 500 cycles. The assembled practical pouch cell achieves an impressive energy density of 372 Wh kg^–1^. This work offers valuable insights into developing high-energy-density solid-state Na–S batteries for practical applications.

## Graphical abstract

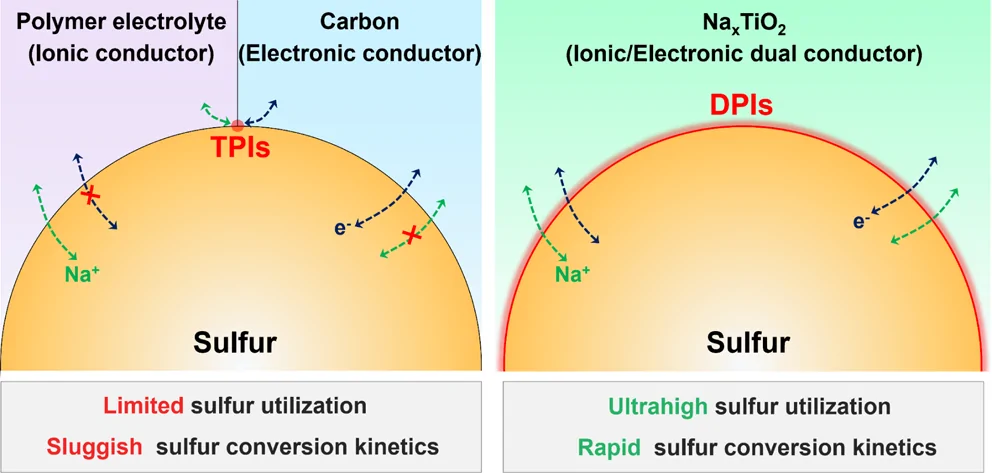

The development of advanced electrochemical energy storage systems has become imperative to meet the burgeoning demands of emerging applications, including wearable electronics, electric vehicles, and smart grids. Transitioning from conventional liquid electrolytes to solid-state counterparts is widely recognized as a pivotal avenue for realizing practical devices with heightened energy density and improved safety.^1–5^ Quasi-solid-state room-temperature sodium–sulfur (Na–S) batteries combine the intrinsic advantages of Na–S chemistry, including low cost, a high theoretical specific capacity (1675 mAh g^–1^), and an exceptional theoretical energy density (1274 Wh kg^–1^), with the merits of quasi-solid polymer electrolytes, such as a high ionic conductivity (10^–4^–10^–3^ S cm^–1^ at room temperature), good interface compatibility, and high safety, thereby holding great promise for next-generation high-energy rechargeable batteries.^6–9^ However, electrolyte solidification substantially reduces liquid-phase ion transport channels, leading to severely sluggish dynamics of ion transfer within the electrode architecture. In addition, the sulfur redox reaction (SRR) inherently involves a sluggish solid–liquid–solid phase transformation, the reaction kinetics of which are further aggravated in a quasi-solid-state environment. To address these challenges, increasing the proportion of conductive carbon and solid electrolyte is commonly adopted to enlarge the sulfur–carbon-electrolyte triple-phase interfaces (TPIs), thereby facilitating charge transport within the cathode.^10–15^ Nevertheless, these electrochemically inactive components significantly compromise the practical energy density of the battery, hindering its competitiveness against existing Na-ion batteries. Particularly under practical conditions featuring a high sulfur content (50 wt %), the dual limitation of ionic and electronic transport severely restricts sulfur utilization, leaving a substantial fraction of active material electrochemically inaccessible and resulting in poor overall performance.^12,13^ Therefore, maximizing sulfur utilization within constrained TPIs and accelerating the efficient catalytic conversion of polysulfides (NaPSs) generated during the SRR remain critical bottlenecks to be resolved for quasi-solid-state Na–S batteries.

Introducing metal compounds with highly polar surfaces as catalytic hosts for sulfur is one of the most common strategies to enhance NaPS conversion kinetics.^16–18^ An ideal electrocatalyst should simultaneously possess two critical attributes: (i) moderate adsorption strength, as dictated by the Sabatier principle, where insufficient adsorption fails to suppress the shuttle effect, while overly strong adsorption hinders the subsequent conversion of NaPS intermediates; (ii) excellent electronic conductivity, which facilitates efficient electron transfer and expands the reaction domains for the SRR, ensuring that the adsorption and conversion of NaPSs can occur directly on the electrocatalyst surface.^10,19–21^ Extensive efforts, including vacancy engineering and heteroatom doping, have been devoted to modulating the electronic structure of metal compounds to enhance intrinsic conductivity and NaPSs’ adsorption strength.^22–24^ However, most reported electrocatalysts are originally designed for conventional liquid-electrolyte systems, where optimization primarily focuses on the adsorption of soluble long-chain NaPSs (Na_2_S_*n*_, 4 ≤ *n* ≤ 8) and their liquid–liquid/liquid–solid conversion processes, while the kinetically sluggish conversion of short-chain NaPSs (Na_2_S_*n*_, 1 ≤ *n* ≤ 3) remains inadequately regulated. In particular, the solid–solid conversion from Na_2_S_2_ to Na_2_S is widely acknowledged as the rate-determining step of the SRR, which impedes the complete sulfur reduction during discharge and ultimately leads to a substantial loss in achievable specific capacity.^25–27^ This limitation is further exacerbated in quasi-solid-state counterparts, where the absence of liquid electrolyte wetting renders solid–solid conversion highly dependent on coupled ionic and electronic transport at the reaction interface. Conventional electrocatalysts typically provide electronic conductivity only, while lacking effective ionic transport capability, and therefore remain unable to resolve the core pain point of limited TPIs.

Herein, we propose the concept of an ion/electron dual-conductive (IEDC) electrocatalyst for quasi-solid-state Na–S batteries. A presodiated TiO_2_ (Na_*x*_TiO_2_) electrocatalyst with IEDC characteristics is fabricated via an electrochemical ion-intercalation method, which extends the active surface from confined TPIs to spacious S–Na_*x*_TiO_2_ dual-phase interfaces (DPIs), enabling rapid catalytic conversion of NaPSs directly at the DPIs (Figure 1a). Our findings demonstrate that the intercalated Na serves as an electron donor to modulate the local coordination environment and electronic structure of TiO_2_, thus enhancing its catalytic activity, which follows a distinct “volcano-shaped” evolution trend as a function of Na content (*x*). At an optimal intercalation concentration (*x* = 0.25), Na_*x*_TiO_2_ markedly accelerates the NaPS conversion process and effectively promotes the completion of the rate-determining step (Na_2_S_2_ to Na_2_S), thereby enhancing sulfur utilization and reaction reversibility. As a result, in a poly­(vinylidene fluoride-*co*-hexafluoropropylene) (PVDF-HFP)-based quasi-solid-state Na–S battery, the Na_0.25_TiO_2_-catalyzed sulfur cathode delivers an impressive initial discharge capacity of 1391.2 mAh g^–1^ at 0.2C even under a high sulfur content of 70 wt %, corresponding to an ultrahigh sulfur utilization of 83.1%, and exhibits superior cycling stability with a mere 0.061% capacity decay per cycle over 500 cycles. Remarkably, the Na_0.25_TiO_2_ electrocatalyst maintains robust performance under a high sulfur mass loading of 3.0 mg cm^–2^ and in a large-scale 7.5 × 8.5 cm^2^ pouch cell, which achieves an impressive cell-level energy density of 372 Wh kg^–1^ and operates stably for 50 cycles, overcoming the limitation where electrocatalysts developed in most works fail to perform effectively in practical devices. This work provides a viable strategy for developing efficient SRR electrocatalysts and represents a significant step toward the practical application of high-performance quasi-solid-state Na–S batteries.

**Figure 1:**
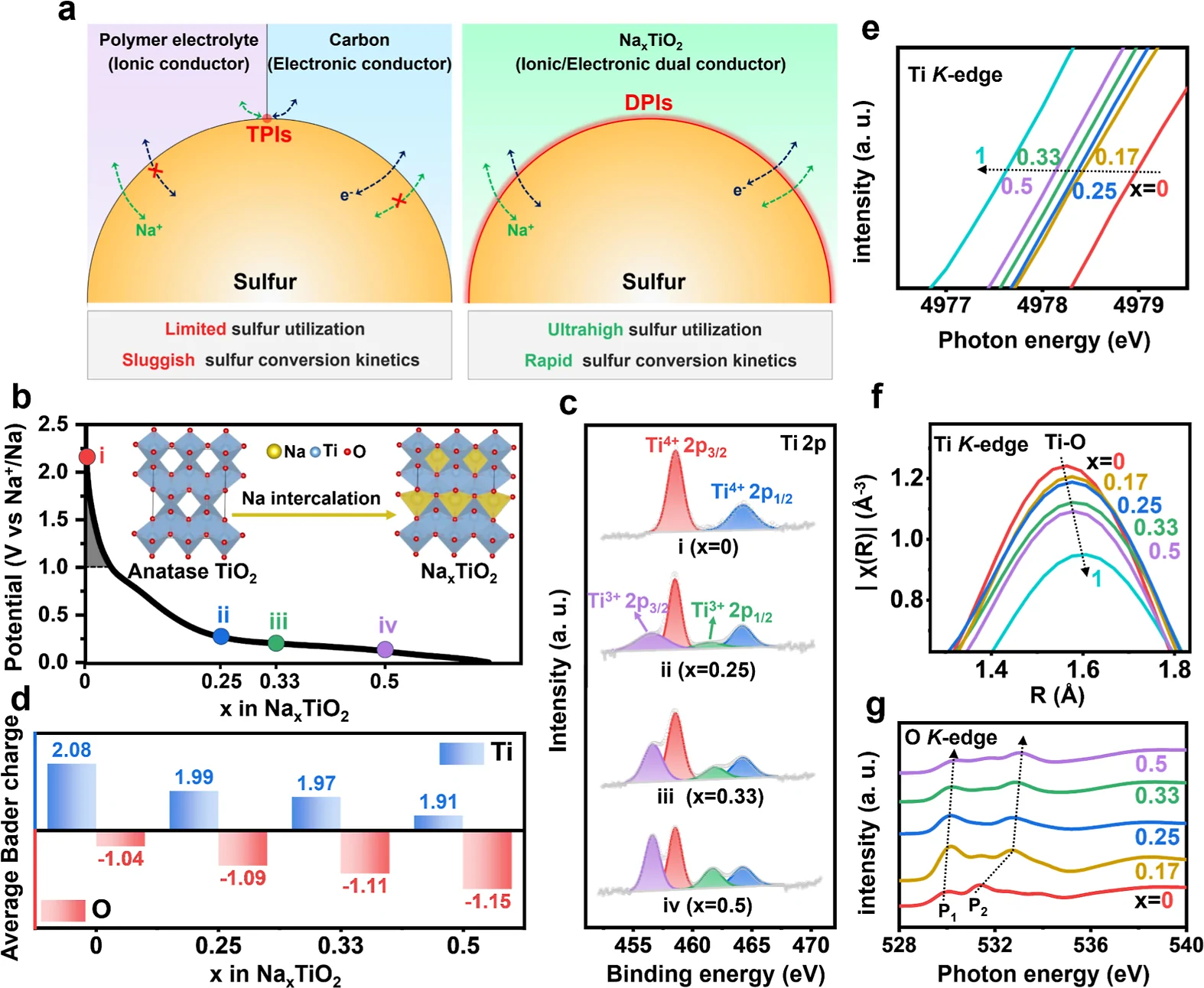
Continuous electrochemical Na-intercalation for regulating the local coordination environment and electronic structure of TiO_2_. (a) Schematic illustration of the sulfur redox reaction (SRR) area at the traditional triple-phase interfaces (TPIs) and dual-phase interfaces (DPIs) constructed by the ion-electronic dual-conductive electrocatalysts. (b) Galvanostatic discharge profile of anatase TiO_2_ during electrochemical Na intercalation, with the inset illustrating the schematic structural evolution from pristine TiO_2_ to Na_*x*_TiO_2_. (c) High-resolution Ti 2p XPS spectra of pristine TiO_2_ and Na_*x*_TiO_2_. (d) Charge variations of Ti and O atoms based on Bader charge analysis. (e,f) Enlarged Ti *K*-edge XANES (e) and FT-EXAFS (f) spectra of pristine TiO_2_ and Na_*x*_TiO_2_. (g) O *K*-edge sXAS spectra of pristine TiO_2_ and Na_*x*_TiO_2_.

## Results and Discussion

A series of Na_*x*_TiO_2_ (0 < *x* < 1) solid solution phases were synthesized via electrochemical Na intercalation into commercial anatase TiO_2_ nanoparticles. The galvanostatic discharge profile in Figure 1b shows a broad discharge plateau region (ii–iv), indicative of the gradual formation of intermediate Na_*x*_TiO_2_ phases during the stepwise sodiation of anatase TiO_2_.^28^ The inset of Figure 1b illustrates the structural evolution from pristine anatase TiO_2_ to the sodiated Na_*x*_TiO_2_ phases, where Na^+^ ions are intercalated into the octahedral interstices coordinated by six surrounding oxygen atoms.^29^ This intercalation occurs with minimal lattice expansion (Figure S1), as also corroborated by the XRD patterns, where the diffraction peaks exhibit a slight overall shift toward lower angles (Figure S2). It should be noted here that TiO_2_ stores Na^+^ ions via a pseudocapacitive rather than an intercalation-based mechanism when the discharge is limited to a cutoff voltage of 1.0 V; namely, no Na^+^ ion intercalation occurs within the TiO_2_ lattice above this potential (the gray area in Figure 1b).^28,29^ Figure S3 shows energy-dispersive X-ray spectroscopy (EDS) spectra corresponding to the points i–iv along the sodiation curve. A progressive increase in the Na signal intensity confirms a gradual rise in Na concentration within the TiO_2_ host structure. X-ray photoelectron spectroscopy (XPS) analysis was conducted to further quantitatively evaluate the Na concentration in the Na_*x*_TiO_2_ phases (Figure 1c). The Ti 2p spectrum of pristine TiO_2_ exhibits two characteristic peaks at binding energies of 458.5 and 464.2 eV, corresponding to Ti 2p_3/2_ and Ti 2p_1/2_ of Ti^4+^, respectively, which confirms the phase purity of the anatase structure.^30^ After a period of Na^+^ intercalation (ii), a partial reduction of Ti^4+^ to Ti^3+^ is observed.^28^ This reduction originates from the fact that Na^+^ intercalation introduces electrons into the TiO_2_ lattice, which occupy the Ti 3d orbitals and result in the formation of Ti^3+^ species for charge compensation. The fitted XPS spectrum shows a Ti^3+^ to Ti^4+^ ratio of approximately 0.33, which correlates with the Na_0.25_TiO_2_ formation (calculation details in Table S1). Upon further discharge (iii, iv), this ratio increases to around 0.5 and 1, corresponding to Na_0.33_TiO_2_ and Na_0.5_TiO_2_, respectively. Bader charge analysis was performed to gain insight into the electronic structure and interatomic interactions upon Na intercalation. Figure S4 depicts the charge density distribution of Na_*x*_TiO_2_ on the (101) crystal facet. Bader charge analysis demonstrates that both Ti and Na atoms carry positive charges (Figures 1d and S5), confirming that Na acts as an electron donor. The donated electrons of a Na atom slightly increase as the Na concentration increases, while the donated electrons of Ti gradually decrease, indicating a reduction in the oxidation state of Ti cations (consistent with the XPS results). Moreover, the electron transfer from Na to O leads to the formation of local Ti–O–Na structural units, which weakens the covalency of the Ti–O bonds. Consequently, this leads to a notable enrichment of electron density around the O atoms, as evidenced by the average Bader charge of O decreasing from −1.04 to −1.15. This change is expected to influence, to some extent, the catalytic activity of TiO_2_ toward SRR, as will be further discussed later.

To further investigate the local bonding evolution of TiO_2_ during continuous electrochemical Na intercalation, X-ray absorption near-edge structure (XANES) and Fourier-transformed extended X-ray absorption fine structure (FT-EXAFS) analyses were performed at the Ti *K*-edge. The Ti *K*-edge XANES spectra (Figures S6 and 1e) display identical spectral features for TiO_2_ before and after sodiation, with peaks P_1_, P_2_, and P_3_ representing the characteristic signatures of TiO_2_.^31^ As the Na concentration increases, a slight shift toward lower energy is observed in the pre-edge region (4976–4979 eV), which is typically regarded as evidence of Ti^4+^ reduction. FT-EXAFS spectra (Figures S7 and 1f) show that continuous Na intercalation induces a drop in peak intensity alongside a shift to a higher radial distance for the first Ti–O coordination shell at ∼1.55 Å. This bond elongation originates from the larger ionic radius of Ti^3+^ (0.67 Å) compared to Ti^4+^ (0.605 Å), while the decreased peak intensity reflects heightened structural disorder caused by mixed Ti^4+^/Ti^3+^ valence states and Na-induced local lattice strain.^31^ Moreover, peaks P_1_ and P_2_ corresponding to O 1s core electron excitations into hybridized O 2p-Ti 3d t_2g_ and O 2p-Ti 3d e_g_ states, respectively, exhibit a distinct shift toward higher energies due to Na intercalation in the O *K*-edge soft X-ray absorption spectra (sXAS, Figure 1g),^32^ which provides further evidence of reduced Ti–O bond covalency and a decrease in Ti^4+^ concentration.

To gain in-depth insight into the regulatory role of Na intercalation on the electrocatalytic behavior of TiO_2_, density functional theory (DFT) calculations were performed to evaluate the adsorption of NaPSs with varying chain lengths Na_2_S_*n*_ (*n* = 1, 2, 4, 6, and 8) on Na_*x*_TiO_2_ substrates (*x* = 0, 0.17, 0.25, 0.33, and 0.5). As clearly evidenced in Figure 2a, Na_*x*_TiO_2_ exhibits significantly enhanced adsorption toward NaPSs compared to pristine TiO_2_, and the binding energy increases with increasing intercalated Na concentration. Differential charge density distributions (Figure 2b) further elucidate the differences in the interactions of NaPSs adsorbed on pristine TiO_2_ and Na_*x*_TiO_2_ at the microscopic electronic level. Compared with pristine TiO_2_, the interfaces between NaPSs and Na_*x*_TiO_2_ (taking *x* = 0.25 as an example) exhibit more pronounced charge accumulation (yellow regions) and depletion (cyan regions), which signifies the formation of robust chemical bonding (chemisorption) between Na_*x*_TiO_2_ and NaPSs. The results confirm that intercalated Na effectively activates the electronic states on the TiO_2_ substrate surface, achieving robust chemical anchoring of NaPSs, which is expected to suppress the shuttle effect and facilitate the nucleation and growth kinetics of Na_2_S during the discharge process. The adsorption efficacy of Na_*x*_TiO_2_ toward NaPSs is visually demonstrated through static adsorption experiments combined with ultraviolet–visible (UV–vis) absorption spectroscopy (Figure 2c and its inset). Initially, a freshly prepared Na_2_S_6_ solution with a deep brownish-yellow color is used as the blank control. Subsequently, equal masses of pristine TiO_2_ and Na_*x*_TiO_2_ with different Na concentrations are separately added to the solution, followed by standing overnight. As observed, the solutions containing Na_*x*_TiO_2_ gradually fade and eventually become colorless, indicating that Na_2_S_6_ is almost completely adsorbed. In contrast, the solution containing pristine TiO_2_ shows only a slight color change, which reflects its markedly weaker adsorption capability toward NaPSs (inset of Figure 2c). Considering the possible influence of residual carbon (acetylene black) introduced during the preparation of Na_*x*_TiO_2_, the Na_2_S_6_ adsorption test of carbon was also conducted, which shows a negligible adsorption capability of carbon (Figure S8). UV–vis absorption spectroscopy was performed on the above solutions to examine the changes in Na_2_S_6_ absorbance induced by different adsorbents (Figure 2c). For the solutions treated with Na_*x*_TiO_2_, the characteristic absorption peak of S_6_^2–^ at ∼420 nm is almost undetectable (enlargement of Figure 2c),^33,34^ while a distinct absorption peak is still observed in the case of pristine TiO_2_.

**Figure 2:**
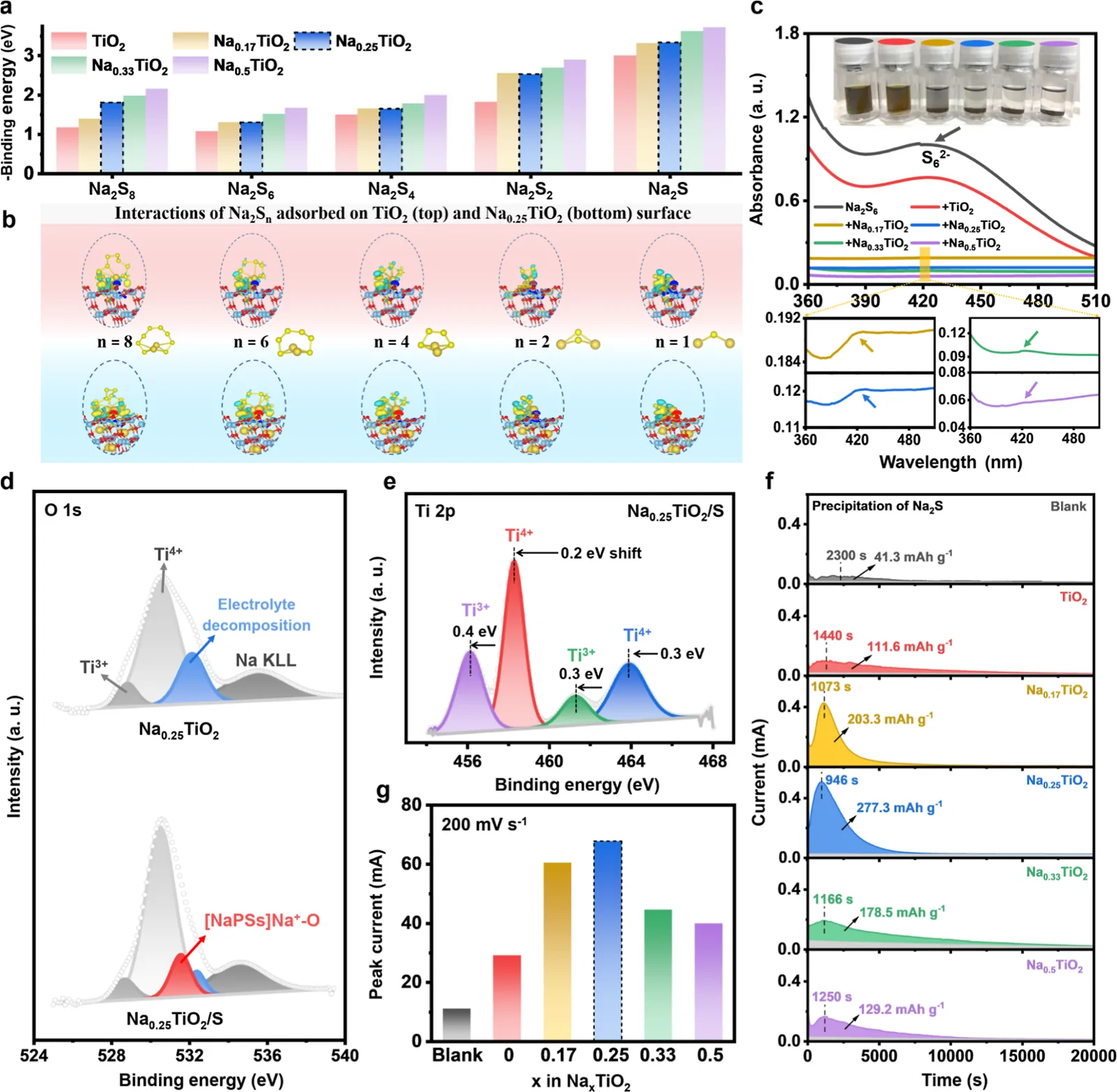
NaPS adsorption and catalytic conversion performance of Na_*x*_TiO_2_ in a liquid electrolyte. (a) Calculated binding energies of Na_2_S_*n*_ (*n* = 1, 2, 4, 6, 8) on TiO_2_ and Na_*x*_TiO_2_ substrates. (b) Differential charge density distributions of Na_2_S_*n*_ adsorbed on TiO_2_ and Na_0.25_TiO_2_ surfaces. (c) UV–vis absorption spectra of Na_2_S_6_ solutions after adsorption. The inset of (c) is the digital photographs of static adsorption experiments (Na_2_S_6_). (d) High-resolution O 1s XPS spectra of pristine Na_0.25_TiO_2_ and the fully discharged Na_0.25_TiO_2_/S cathode. (e) High-resolution Ti 2p XPS spectra of the fully discharged Na_0.25_TiO_2_/S cathode. (f) Potentiostatic nucleation curves of Na_2_S on TiO_2_ and Na_*x*_TiO_2_. (g) Peak current comparisons of TiO_2_ and Na_*x*_TiO_2_ in symmetrical CV curves at 200 mV s^–1^.

XPS further identifies the adsorption sites responsible for the strong interactions between Na_*x*_TiO_2_ (taking *x* = 0.25 as an example) and NaPSs (Figure 2d). In the O 1s spectrum of pristine Na_0.25_TiO_2_, the peaks at 528.8 and 530.5 eV correspond to two Ti–O bonding environments, namely, Ti^3+^–O and Ti^4+^–O, confirming again that Na intercalation induces the formation of Ti^3+^. The peak at 532.3 eV is assigned to C–O/H–O species representing organic/inorganic components derived from ether-based electrolyte decomposition.^31^ In contrast, a new peak appears at 531.6 eV in the O 1s spectrum of the sulfur cathode incorporating the Na_0.25_TiO_2_ electrocatalyst (Na_0.25_TiO_2_/S) at the discharged state. This feature can be attributed to Na–O interactions formed as a result of NaPSs being adsorbed on the Na_0.25_TiO_2_ surface, where Na and O originate from NaPSs and Na_0.25_TiO_2_, respectively. This result indicates that the electrochemical ion-intercalation process effectively modulates the TiO_2_ electronic structure by introducing Na^+^ into the TiO_2_ lattice, which increases the electron density around the O atoms (as discussed in Figure 1d). As a consequence, the O atoms exhibit enhanced Lewis basicity, enabling them to capture Na^+^ Lewis acidic species from NaPSs, which form robust Na–O bonds and thus achieve strong chemisorption of sulfur species. Additionally, in the Ti 2p spectrum, all four peaks corresponding to Ti 2p_1/2_ and Ti 2p_3/2_ exhibit a slight shift of approximately 0.2–0.4 eV toward lower binding energies in Na_0.25_TiO_2_/S (Figure 2e), further indicating pronounced interfacial interaction associated with electron transfer from Na_2_S to Na_0.25_TiO_2_.

The liquid–solid phase transition involving Na_2_S nucleation is widely regarded as the pivotal step governing capacity contribution during the discharge process. Accordingly, the catalytic performance of Na_*x*_TiO_2_ toward Na_2_S precipitation was evaluated, with the corresponding capacity calculated based on Faraday’s law (Figure 2f).^35^ It is evident that cells incorporating the Na_*x*_TiO_2_ electrocatalysts exhibit significantly higher precipitation capacities and earlier nucleation times than both the blank control and the pristine TiO_2_ counterparts. Notably, the catalytic performance follows a “volcano-shaped” trend as a function of the Na intercalation concentration (*x*). The cell with Na_*x*_TiO_2_ delivers the highest capacity of 277.3 mAh g^–1^ and the earliest time of 946 s when *x* reaches 0.25. However, performance degradation is observed as *x* increases to 0.33, and at *x* = 0.5, the capacity decreases by more than half to 129.2 mAh g^–1^, with a 304 s delay in nucleation time. Symmetric cyclic voltammetry (CV) tests further corroborate this trend (Figure S9), where the Na_0.25_TiO_2_-based symmetric cell shows the highest redox current response (67.8 mA, Figure 2g). These results suggest that stronger adsorption strength does not necessarily translate to superior catalytic activity, suggesting the involvement of other underlying factors that will be discussed in detail later.

The superior catalytic activity of Na_0.25_TiO_2_ motivates us to evaluate its potential in PVDF-HFP-based quasi-solid-state Na–S batteries. Figure 3a compares the CV profiles of quasi-solid-state Na–S batteries without a catalyst and with different electrocatalysts incorporated into the sulfur cathode, all of which exhibit similar electrochemical behaviors. Two pronounced reduction peaks located at 2.1 and 1.7 V (denoted as R_1_ and R_2_) are observed, together with two oxidation peaks centered at 2.0 and 2.4 V (denoted as O_1_ and O_2_), which can be ascribed to a series of complex phase-transition processes involving long-chain (S_8_ → Na_2_S_*n*_, 4 ≤ *n* ≤ 8) and short-chain (Na_2_S_*n*_ → Na_2_S, 1 ≤ *n* ≤ 4) NaPS intermediates. Compared with the catalyst-free cathode and those containing other electrocatalysts, the Na_0.25_TiO_2_/S cathode exhibits the highest redox peak intensities and the smallest separation between the reduction and oxidation peaks, indicative of a higher reversible capacity and significantly accelerated SRR kinetics. It has been mentioned above that no further Na^+^ ion intercalation occurs within the bulk of Na_*x*_TiO_2_ when the discharge cutoff voltage is set to 1.0 V. Moreover, no additional oxidation peaks associated with the deintercalation of intercalated Na^+^ ions are observed during the charging process in comparison with the CV profiles of the Blank/S and TiO_2_/S cathodes. This indicates that Na_*x*_TiO_2_, serving as an electrocatalyst, maintains a robust structural phase stability within the operating voltage window (1.0–2.8 V) of Na–S batteries. Such stability is attributed to the formation of rigid Ti–O–Na local structural units within the Na_*x*_TiO_2_ bulk during the electrochemical preintercalation process, resulting in a thermodynamically stable phase. These Na^+^ ions are firmly pinned within the lattice and behave as electrochemically inert species during the SRR. The Tafel slopes (Figure S10) of the reduction peaks (R_1_, R_2_) and oxidation peaks (O_1_, O_2_) were obtained from the CV profiles and further elucidate that the Na_0.25_TiO_2_ electrocatalyst possesses bifunctional catalytic activity, which effectively accelerates the SRR kinetics and thereby holds promise for enhancing the cycling performance of quasi-solid-state Na–S batteries.

**Figure 3:**
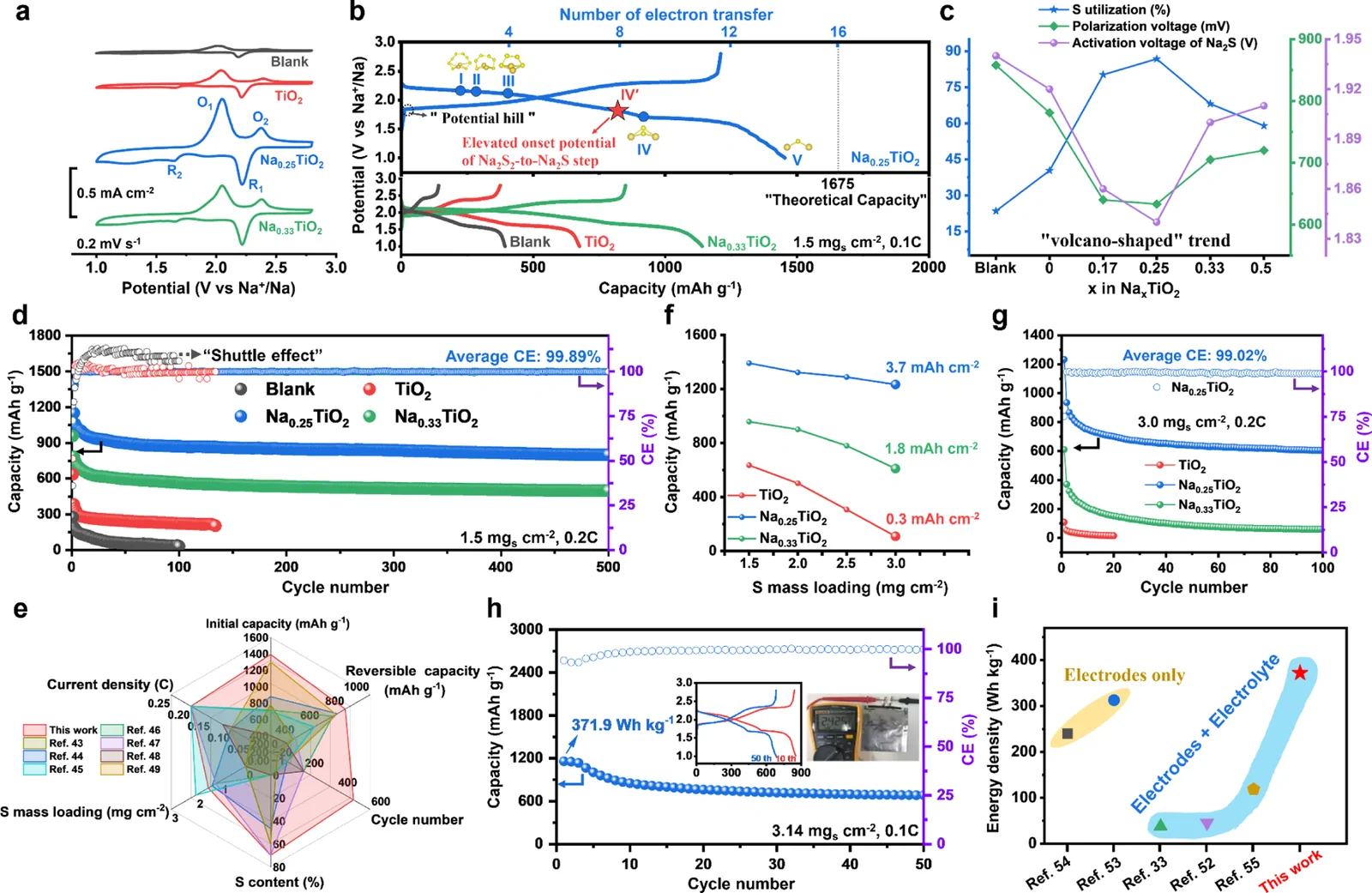
Electrochemical performance of Na_*x*_TiO_2_ electrocatalysts in quasi-solid-state Na–S batteries. (a) CV curves of the cathodes without or with different electrocatalysts. (b) Galvanostatic discharge–charge profiles at 0.1C. (c) Statistical “volcano-shaped” trends of S utilization, polarization voltage, and Na_2_S activation voltage versus Na concentration. (d) Long-term cycling performance at 0.2C. (e) Comparison of electrochemical performance with previously reported works in (coin-type) quasi-solid-state Na–S batteries using a polymer electrolyte. (f) Discharge capacity variations under different S mass loading at 0.2C. (g) Cycling stability under a high S mass loading (3.0 mg cm^–2^) at 0.2C. (h) Cycle performance of the pouch cell at 0.1C. (i) Comparison of energy densities with previously reported works in Na–S pouch cells. Note: “Electrodes + Electrolyte” means that the energy density is calculated based on the mass of the electrodes (anodes and cathodes) and electrolyte.

Figures 3b and S11 present the galvanostatic discharge–charge profiles of the Na_0.25_TiO_2_/S cathode and the control electrodes. All curves display two well-defined voltage plateaus during both discharge and charge processes, which correlate well with the two reduction and two oxidation peaks observed in the CV profiles. The voltage plateau at 2.16–2.21 V is attributed to the solid–liquid transition from S to Na_2_S_8_ (where the point I on the blue curve denotes the end of this stage), followed by the formation of Na_2_S_6_ and Na_2_S_4_ at 2.10–2.16 V (points II and III).^36–39^ The sloping region between 1.70 and 2.10 V is associated with the liquid–solid phase transition from Na_2_S_4_ to Na_2_S_2_ (point IV), while the voltage plateau at 1.62–1.70 V and the subsequent sloping region (IV → V) represent the further conversion from Na_2_S_2_ to Na_2_S.^38,39^ Notably, the discharge and charge plateaus are substantially prolonged for all sulfur cathodes catalyzed by the Na_*x*_TiO_2_ series. Among them, the Na_0.25_TiO_2_/S cathode delivers the highest initial discharge capacity of 1453.9 mAh g^–1^ at 0.1C (1C = 1675 mA g^–1^), corresponding to an exceptionally high S utilization of 86.8%, which represents ∼3.7 and ∼2.2× higher than those of the Blank/S (393.3 mAh g^–1^, 23.5%) and TiO_2_/S (675.7 mAh g^–1^, 40.3%) cathodes, respectively. More impressively, considering the overall 16-electron-transfer reaction of Na–S batteries (S_8_ + 16e^–^ + 16Na^+^ ↔ 8Na_2_S, 1675 mAh g^–1^), the Na_0.25_TiO_2_/S electrode realizes the 100% capacity associated with the conversion of S_8_ to Na_2_S_2_ (S_8_ + 8e^–^ + 8Na^+^ → 4Na_2_S_2_, 837.5 mAh g^–1^). In fact, the Na_0.25_TiO_2_/S electrode already delivers a discharge capacity of 913 mAh g^–1^ upon discharging to 1.7 V (point IV), which indicates that Na_0.25_TiO_2_ effectively advances the onset potential of the kinetically most sluggish SRR step (Na_2_S_2_ → Na_2_S) to a higher 1.8 V (point IV′). Consequently, a capacity of 616.4 mAh g^–1^ is delivered during this stage (IV → V), accounting for as much as 73.7% of the total capacity theoretically available from the complete Na_2_S_2_-to-Na_2_S conversion (4Na_2_S_2_ + 8e^–^ + 8Na^+^ → 8Na_2_S, 837.5 mAh g^–1^). In sharp contrast, the Blank/S and TiO_2_/S electrodes achieve only 25% and 34.6% of this capacity, respectively, while other Na_*x*_TiO_2_/S cathodes elevate this ratio to over 50%. These findings demonstrate the superior catalytic proficiency of Na_*x*_TiO_2_ in facilitating both the adsorption-conversion of long-chain NaPSs and the challenging solid-to-solid conversion of short-chain NaPSs. Moreover, owing to the intrinsically poor electronic and ionic conductivity of Na_2_S, its phase nucleation back to NaPSs is inherently difficult, typically requiring a high charging cutoff voltage (“potential hill”) to overcome the barrier for Na extraction.^39–41^ In contrast, the Na_0.25_TiO_2_/S electrode exhibits a significantly lower activation voltage of 1.84 V and a reduced charging voltage plateau, further evidencing the superior reverse catalytic activity of Na_0.25_TiO_2_ toward the sulfur oxidation reaction. Consequently, the Na_0.25_TiO_2_ electrode delivers the smallest polarization voltage of 633 mV throughout the complete S_8_ ↔ Na_2_S redox cycle. A statistical summary of these performance indicators is provided in Figure 3c, from which it can be seen that sulfur cathodes employing different Na_*x*_TiO_2_ electrocatalysts exhibit pronounced differences in sulfur utilization, polarization voltage, and Na_2_S activation voltage. Notably, these metrics display a “volcano-shaped” dependence on the increased Na intercalation concentration (*x*), which is fully consistent with the previously observed patterns. It should be noted that although Na_*x*_TiO_2_ exhibits pseudocapacitive Na^+^ storage above 1.0 V, this process cannot be responsible for the enhanced sulfur conversion kinetics. Pristine TiO_2_, which also undergoes similar Na^+^ storage behavior within the Na–S battery operating window, shows much inferior catalytic performance compared with Na_*x*_TiO_2_. The composition-dependent catalytic activity among different Na_*x*_TiO_2_ further confirms that the bulk Na intercalation-induced Ti–O coordination environment and electronic structure, rather than pseudocapacitive Na^+^ behavior, are responsible for the accelerated sulfur conversion reactions, as will be further discussed later.

As expected, the Na_0.25_TiO_2_/S cathode exhibits outstanding long-term cycling performance, which delivers a high initial discharge capacity of 1391.2 mAh g^–1^ and retains a reversible capacity of 800.3 mAh g^–1^ after 500 cycles at 0.2C (Figure 3d), corresponding to an exceptionally low capacity decay rate of 0.061% per cycle (based on the second cycle). In sharp contrast, the Blank/S and TiO_2_/S electrodes exhibit rapid failure, with capacities dropping to a mere 31.8 and 211.3 mAh g^–1^ after just over 100 cycles, respectively. Although the Na_0.33_TiO_2_-catalyzed sulfur cathode also exhibits relatively good cycling stability, its capacity after 500 cycles reaches only 62.3% of that delivered by the Na_0.25_TiO_2_/S cathode. Moreover, the Na_0.25_TiO_2_/S electrode achieves a high initial Coulombic efficiency (CE) of 81.0% and an average CE of 99.89%, indicative of highly efficient catalytic conversion of sulfur species. By comparison, the Blank/S and TiO_2_/S electrodes exhibit a much lower initial CE of 36.0% and 51.2%, respectively, followed by abnormal CE exceeding 100%. This phenomenon is attributed to the severe shuttle effect of NaPSs,^42^ which triggers continuous parasitic reactions with the Na metal anode and results in the failure of the charging voltage to rise, reflecting the weak adsorption capability of pristine TiO_2_ for NaPSs. The Na_0.25_TiO_2_/S cathode exhibits electrochemical performance in quasi-solid-state Na–S batteries that is markedly superior to that reported previously (Figure 3e and details in Table S2).^43–49^

Moreover, a high gravimetric capacity does not necessarily translate into a high areal capacity, the latter being a critical metric for enhancing the practical energy density of batteries.^50^ Therefore, the performance of the Na_0.25_TiO_2_/S cathode was further evaluated under high sulfur mass loading in thick electrodes. When the sulfur mass loading is doubled from 1.5 to 3.0 mg cm^–2^, the Na_0.25_TiO_2_/S cell still delivers a high initial discharge capacity of 1233.4 mAh g^–1^, retaining 88.7% of the capacity obtained at the lower sulfur mass loading. Correspondingly, the areal capacity is markedly increased from 2.1 to 3.7 mAh cm^–2^ (Figure 3f). This value presents a significant advantage over current sodium-ion batteries that predominantly utilize sodium vanadium phosphate (NVP) cathodes. To achieve a comparable areal capacity, an NVP-based electrode would require an active material loading of 31.62 mg cm^–2^ (implying a total electrode loading of at least 40 mg cm^–2^), even assuming it reaches its theoretical capacity (117 mAh g^–1^).^51^ Such extreme loading requirements impose severe challenges on internal ion/electron transport pathways, long-term structural integrity, and industrial-scale electrode fabrication processes, making this an almost unattainable target for current sodium-ion batteries. In sharp contrast, the TiO_2_/S cathode suffers from pronounced capacity decay as the sulfur mass loading increases, which delivers a low areal capacity of only 0.3 mAh cm^–2^ (108.3 mAh g^–1^). Although the Na_0.33_TiO_2_/S electrode achieves an areal capacity of 1.95 mAh cm^–2^ at a sulfur mass loading of 2.5 mg cm^–2^, this value decreases to 1.8 mAh cm^–2^ at 3.0 mg cm^–2^, which is less than half that of the Na_0.25_TiO_2_/S electrode. In addition, the Na_0.25_TiO_2_/S cathode maintains a high initial CE of 75.1% and an average CE of 99.02% at 0.2C, while retaining a discharge capacity exceeding 600 mAh g^–1^ after 100 cycles (Figure 3g). In sharp contrast, the Na_0.33_TiO_2_/S electrode retains only 59.7 mAh g^–1^, whereas the TiO_2_/S cathode suffers nearly complete capacity fading after merely 20 cycles. To further evaluate the practical applicability, a pouch cell with dimensions of 7.5 × 8.5 cm^2^ was assembled (Figure 3h), which exhibits a stable open-circuit voltage of 2.43 V, indicative of good electrochemical contact and reliable device encapsulation (inset of Figure 3h). The pouch cell delivers an initial specific discharge capacity of 1158.8 mAh g^–1^ at 0.1C, corresponding to a high energy density of 371.9 Wh kg^–1^ (based on the total mass of all cell components, Table S3). This value is highly competitive among the leading reported performances for Na–S pouch cells to date (Figure 3i and details in Table S4).^33,52–55^ After 50 cycles, the cell retains 60% of its initial capacity (684.1 mAh g^–1^) with a stable average CE exceeding 99.5%. The galvanostatic charge–discharge profiles at the 10th and 50th cycles (inset of Figure 3h) show high overlap, with no discernible increase in voltage polarization.

The Na_0.25_TiO_2_/S cathode also demonstrates superior rate capability (Figures S12 and 4a,b). Upon a 10-fold increase in current density (from 0.1 to 1C), the Na_0.25_TiO_2_/S electrode maintains over 60% of its initial capacity (889.4 mAh g^–1^), with the corresponding voltage polarization increasing by a mere 105 mV (from 615 to 720 mV). In contrast, the Na_0.33_TiO_2_/S and TiO_2_/S cathodes deliver only 42% (480.0 mAh g^–1^) and 14.2% (96.2 mAh g^–1^) of their respective initial capacities, while their voltage polarization surges by 255 mV (690 → 945 mV) and 455 mV (762 → 1217 mV). To further elucidate the exceptional electrochemical performance of Na_0.25_TiO_2_/S compared to Na_0.33_TiO_2_/S and TiO_2_/S in quasi-solid-state Na–S batteries, CV measurements at various scan rates (0.2–0.8 mV s^–1^) were conducted to evaluate the Na^+^ transport kinetics (Figure S13). According to the Randles–Sevcik equation, the peak current (*I*_p_) scales linearly with the square root of the scan rate (ν), enabling the assessment of Na^+^ transport kinetics via the slope (*b*), where *b* is proportional to the Na^+^ diffusion coefficient (*D*_Na_^+^).^56^ As shown in Figure 4c, the *b*-value follows the same “volcano-shaped” trend observed previously as the Na intercalation concentration increases. The Na_0.25_TiO_2_/S cathode delivers significantly higher *b*-values during both sulfur reduction and oxidation reactions than those of the Na_0.33_TiO_2_/S and TiO_2_/S counterparts, which demonstrates that Na_0.25_TiO_2_ possesses superior Na^+^ transport capabilities. Moreover, the electrochemical impedance spectroscopy (EIS) tests were performed to disclose that the Na_0.25_TiO_2_/S cell maintains a stable and remarkably low interfacial resistance throughout extended cycling (Figure 4d). In stark contrast, the interfacial impedance of the TiO_2_/S cell surges to 763.4 Ω after only 150 cycles—a 17-fold increase from its initial state, while the Na_0.33_TiO_2_/S cell also exhibits substantial impedance growth, rising from 33.8 Ω initially to 276.8 Ω at the 200th cycle. To gain deeper insight into the reaction kinetics, temperature-dependent EIS measurements were performed to probe the activation energy (*E*_a_) required for NaPS conversion on different cathodes (Figure 4e). According to the Arrhenius equation, the *E*_a_ for Na^+^ diffusion in the Na_0.25_TiO_2_/S electrode is 18.6 kJ mol^–1^, which is 44.9% and 55.2% lower than the values obtained for Na_0.33_TiO_2_/S (33.8 kJ mol^–1^) and TiO_2_/S (38.9 kJ mol^–1^), respectively. This substantial reduction in *E*_a_ demonstrates that Na_0.25_TiO_2_ possesses significantly enhanced electrocatalytic activity toward facilitating Na_2_S formation. Raman spectroscopy, complemented by XPS analysis, was employed to investigate the Na_0.25_TiO_2_-catalyzed cathode in its fully discharged (1.0 V) and charged (2.8 V) states across different cycles (Figures 4f and S14). As illustrated in Figure 4f, when the cell is first discharged to 1.0 V (DSG@1), a prominent characteristic peak of Na_2_S (S^2–^) is detected at 456 cm^–1^, which confirms that Na_2_S is the final discharge product.^57,58^ Upon charging to 2.8 V (CHG@1), the intensity of the S^2–^ peak sharply diminishes, accompanied by the emergence of a stretching vibration peak at around 200 cm^–1^ assigned to elemental sulfur (S^0^).^57,58^ This observation demonstrates that the majority of Na_2_S is effectively oxidized back to S, corroborating the excellent initial CE of the Na_0.25_TiO_2_/S cathode. During the subsequent 20 charge–discharge cycles, the characteristic peaks of Na_2_S and S exhibit a highly rhythmic alternation of appearance and disappearance, indicating robust electrochemical reversibility of the S-to-Na_2_S conversion. This conclusion is further supported by the high-resolution S 2p XPS spectra collected for the cathode in the discharged and charged states (Figure S14). Ex situ scanning electron microscopy (SEM) was employed to track the morphological evolution of the Na_0.25_TiO_2_/S cathode at different cycling stages (Figure S15). As cycling proceeds, the Na_0.25_TiO_2_-catalyzed cathode exhibits a distinct grain-refinement trend rather than structural collapse or electrochemically inactive sulfur agglomerates.

**Figure 4:**
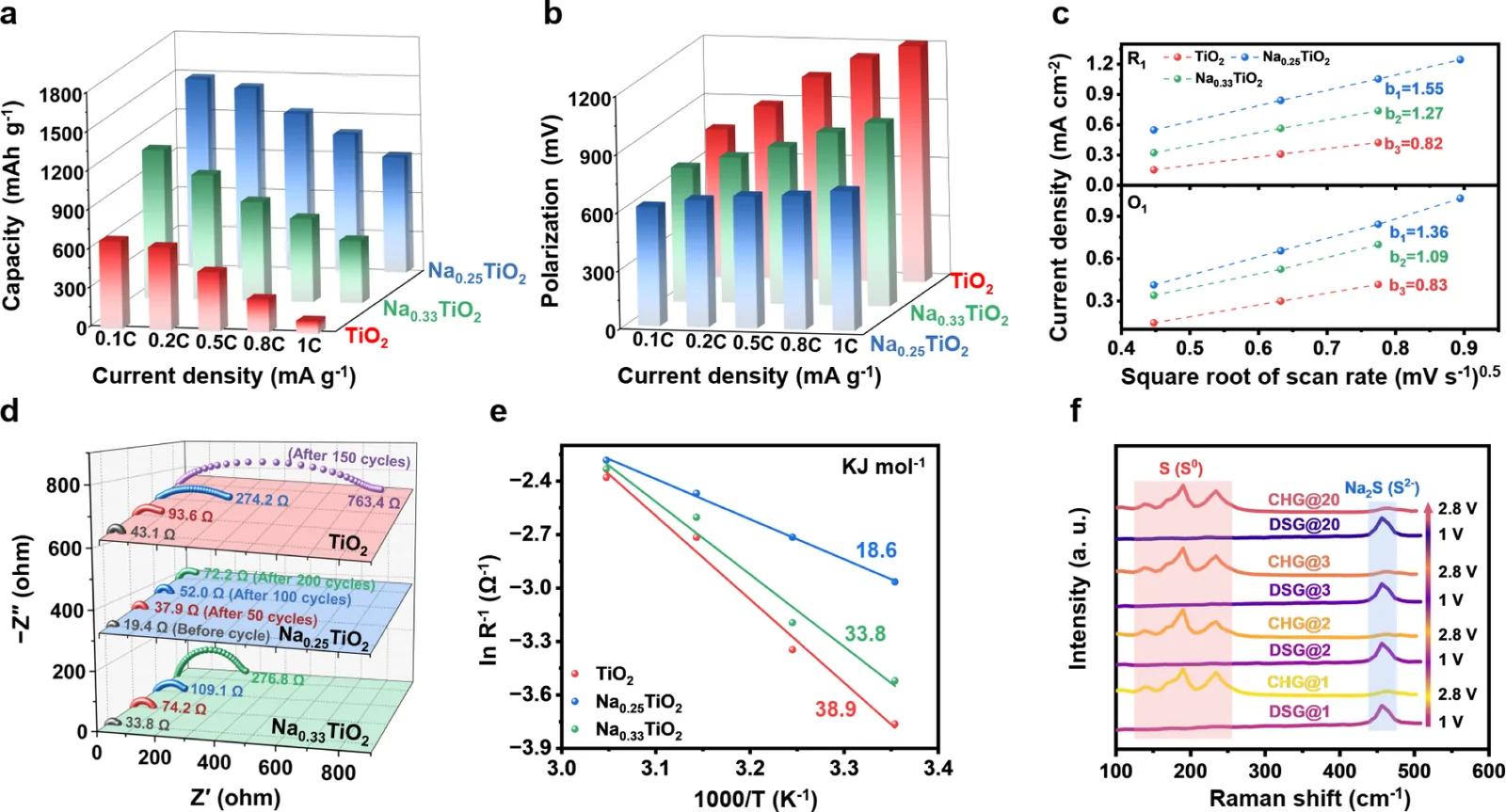
Kinetics and reversibility of Na_*x*_TiO_2_ electrocatalysts in quasi-solid-state Na–S batteries. (a,b) Rate performance of cells with Na_*x*_TiO_2_ electrocatalysts at 0.1–1C. (c) Na^+^ diffusion kinetics fitted by the Randles–Sevcik equation. (d) EIS impedance evolution during cycling. (e) Activation energy (*E*_a_) for NaPS conversion calculated via the Arrhenius equation. (f) Raman spectra of the Na_0.25_TiO_2_/S cathode at different cycling states. The “DSG@1” and “CHG@1” stand for “after the first discharge” and “after the first charge”, respectively.

To gain deeper insight into the underlying mechanism by which the Na intercalation concentration modulates the electronic structure of Na_*x*_TiO_2_ and thereby governs the electrochemical performance, the electronic conductivity of Na_*x*_TiO_2_ (*x* = 0, 0.17, 0.25, 0.33, and 0.5) was systematically analyzed through density of states (DOS) calculations. The computational results clearly demonstrate that Na intercalation profoundly alters the band structure of TiO_2_ (Figure 5a). The valence band of Na_*x*_TiO_2_ is dominated by O 2p orbitals, while the conduction band is mainly contributed by Ti 3d orbitals. For pristine TiO_2_, the DOS at the Fermi level (*E*_F_) is zero, with a calculated band gap of 3.17 eV, which is in close agreement with previously reported experimental values (3.0–3.2 eV).^59^ This electronic structure is consistent with the wide-band gap semiconducting nature of anatase TiO_2_, which is expected to exhibit low intrinsic electronic conductivity in the absence of defects or dopants. As Na^+^ is progressively intercalated into the interstitial sites of the TiO_2_ lattice (0 < *x* ≤ 0.25), a finite and continuous DOS emerges at *E*_F_, indicating a semiconductor-to-metal transition and a corresponding enhancement in electronic conductivity. This behavior is attributed to the role of Na as an electron donor, injecting electrons into the TiO_2_ host and occupying the empty Ti 3d orbitals. However, as the Na concentration further increases (*x* ≥ 0.33), the DOS at *E*_F_ vanishes, accompanied by the formation of a new band gap of 0.6 eV, which reverts to a semiconducting character, leading to a deterioration of electronic conductivity. To elucidate the intrinsic origin of the nonmonotonic influence of Na intercalation concentration on the electronic conductivity of TiO_2_, a comparative analysis of the spin density distributions was performed for Na_*x*_TiO_2_ at low (*x* = 0.25) and high (*x* = 0.5) concentrations. In pristine TiO_2_, the spin-up and spin-down states are symmetric (Figure 5a), yielding zero net magnetic moment, consistent with its nonmagnetic semiconducting nature.^60^ Upon Na^+^ intercalation, spin polarization develops near *E*_F_, indicating that the injected electrons occupy Ti 3d orbitals in an unpaired manner, thereby inducing localized magnetic moments. For Na_0.25_TiO_2_, the calculated total spin magnetic moment is 5.5 μ_B_ (Figure 5b). Notably, the injected electrons by Na donors are not uniformly distributed over all Ti^4+^ sites but are selectively localized on a subset of Ti^4+^ ions (eight sites), corresponding to an average occupation of about 0.7 e^–^ per site. At *x* = 0.5, the total magnetic moment rises markedly to 11.1 μ_B_, with electron localization extending to 12 Ti^4+^ sites and an increased average occupation (0.9 e^–^ per site). This progressive localization of Ti 3d electrons provides a microscopic explanation for the conductivity degradation at high Na concentration.^61^ As the number of localized 3d electrons increases, the enhanced electron–electron Coulomb interaction drives splitting between occupied and unoccupied Ti 3d states, promoting electron localization and reopening the band gap (Figure 5d).

**Figure 5:**
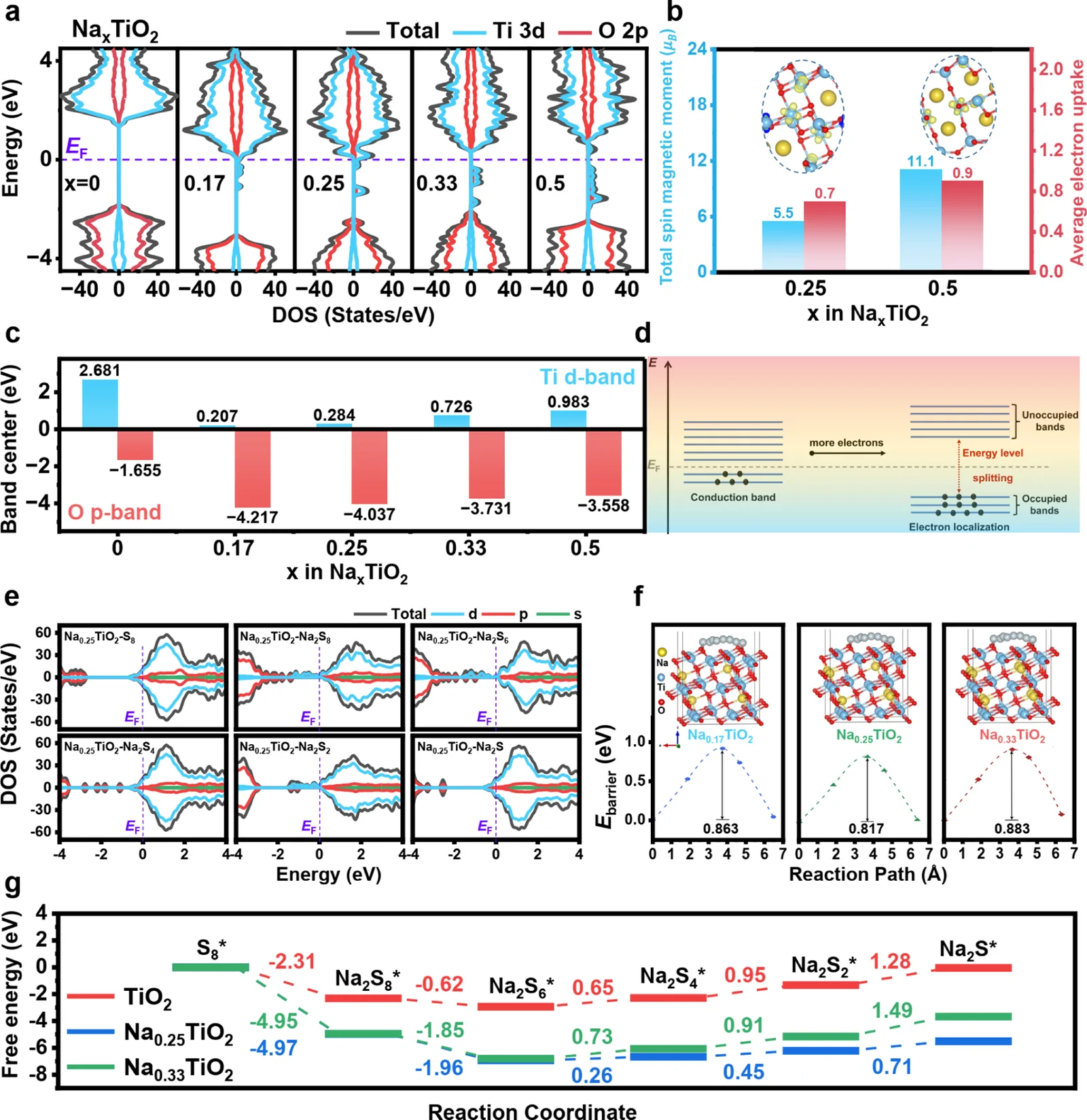
Theoretical insights into the electronic structure and catalytic mechanism of Na_*x*_TiO_2_. (a) DOS of Na_*x*_TiO_2_. (b) Calculated total spin magnetic moments and visualized spin density distributions for Na_0.25_TiO_2_ and Na_0.5_TiO_2_. (c) Evolution of Ti 3d and O 2p band centers (ε_d_ and ε_p_) as a function of Na intercalation concentration. (d) Schematic illustration of the electron localization at high Na concentrations. (e) DOS of Na_0.25_TiO_2_ after the adsorption of various NaPS intermediates. (f) Calculated surface Na^+^ migration paths and corresponding energy barriers for Na_*x*_TiO_2_. (g) Gibbs free energy profiles for the stepwise reduction of S to Na_2_S on TiO_2_ and Na_0.25_TiO_2_ models.

As a pivotal parameter for correlating electronic structures with catalytic activity, the band center is widely adopted as an effective descriptor for predicting the catalytic performance of electrocatalysts.^23^ To elucidate the evolution of catalytic behavior in Na_*x*_TiO_2_, we analyzed the Ti 3d and O 2p band centers (ε_d_ and ε_p_) based on DOS (Figure 5c). Compared to pristine TiO_2_, the introduction of Na induces a significant downward shift of both ε_d_ and ε_p_ toward lower energies, indicating electron injection into the TiO_2_ host and a resulting reconfiguration of the electronic structure. With increasing Na concentration, both band centers exhibit a gradual upshift, reflecting the nonmonotonic dependence on Na content. On the one hand, the position of ε_d_ relative to the *E*_F_ provides insight into electronic transport, as conduction in Na_*x*_TiO_2_ is dominated by Ti 3d states. As *x* increases from 0, ε_d_ shifts toward the *E*_F_, indicating enhanced electron occupation and delocalization of Ti 3d electrons, which facilitates charge transport. At higher Na concentration, however, ε_d_ reversely shifts away from the *E*_F_, consistent with the reduced conductivity. This “approaching-then-receding” behavior of ε_d_ mirrors well the overall “enhancement-then-decline” trend of the system’s conductivity. Although ε_d_ at *x* = 0.17 is closest to *E*_F_ (0.207 eV), the carrier density remains insufficient to establish a continuous conduction network. In contrast, at *x* = 0.25, a higher carrier concentration and increased DOS near *E*_F_ enable the formation of more effective conduction pathways, resulting in optimal electronic conductivity despite a slightly larger ε_d_ (0.284 eV). From the perspective of catalytic interaction, Na_*x*_TiO_2_ exhibits strong affinity toward NaPSs, driven by the robust Na–O interactions, with binding strength increasing monotonically with Na concentration. The evolution of ε_p_ toward *E*_F_ (for *x* ≥ 0.17) is broadly consistent with the band center theory, which associates the higher-lying band centers with enhanced interfacial interactions. However, this paradigm does not apply to pristine TiO_2_. Although its ε_p_ is closest to the *E*_F_, it exhibits weak absorption toward NaPSs. This deviation arises because surface O atoms in pristine TiO_2_ are engaged in strong covalent bonding, limiting the availability of reactive electronic states for interfacial coupling.

Given that the adsorption of sulfur species during cycling can influence interfacial charge transport, the electronic structure of Na_0.25_TiO_2_ after NaPS adsorption was further examined (Figure 5e). All adsorption configurations exhibit finite DOS near the *E*_F_, confirming that the composite system retains its metallic character throughout the SRR. This ensures continuous charge transport across the interface, even during the adsorption of long-chain NaPSs, and thus facilitates the deep reversible sulfur species conversion. To further probe ionic transport, the surface Na^+^ migration behavior on the Na_*x*_TiO_2_ surface was investigated using the climbing-image nudged elastic band (CI-NEB) method. Based on the surface symmetry and previous studies,^62^ four possible adsorption configurations were considered, including one hollow site (A) and three cap sites (B, C, and D), with their top-view and side-view geometries illustrated in Figure S16. The migration path from site A to the adjacent A′ site was constructed for *x* = 0.17, 0.25, and 0.33 (Figure 5f), with six intermediate images interpolated between fully relaxed initial and final states. The calculated diffusion barrier (*E*_barrier_) decreases from 0.863 eV (*x* = 0.17) to 0.817 eV (*x* = 0.25) and then increases to 0.883 eV at *x* = 0.33. Notably, the surface Na^+^ migration pathways and the types of adsorption sites involved in the diffusion process remain identical across all Na concentrations, indicating that the observed differences in diffusion kinetics do not originate from changes in geometric diffusion channels. Instead, this behavior stems from changes in the electronic environment and the corresponding potential energy surface induced by Na intercalation. Moderate Na intercalation introduces extra electrons that redistribute the surface electrostatic potential of TiO_2_, leading to a flattened energy landscape for Na^+^ migration and thus a reduced diffusion barrier. Conversely, excessive Na intensifies local Na^+^ ion–ion repulsion and the resulting enhancement of the potential energy field, which elevates the transition-state energy for Na^+^ migration. Consequently, the modulation of Na^+^ diffusion behavior on the TiO_2_ surface by Na intercalation exhibits distinct nonmonotonic characteristics, where *x* = 0.25 corresponds to the lowest surface migration barrier and the most favorable ionic transport kinetics.

To evaluate the influence of Na_*x*_TiO_2_ on NaPSs conversion kinetics from a thermodynamic perspective, the Gibbs free energy evolution of key intermediates along the stepwise reduction pathway from S to Na_2_S was systematically calculated (Figure 5g). The initial conversion from S_8_ to Na_2_S_8_* and Na_2_S_6_* exhibits significant exothermic characteristics, indicating that the cleavage of long-chain NaPSs occurs spontaneously on all investigated surfaces. However, distinct positive free energy changes emerge during the successive bond-breaking steps from Na_2_S_6_* → Na_2_S_4_* → Na_2_S_2_* → Na_2_S*, which indicates that the further cleavage and ultimate transformation of NaPSs are hindered by significant energy barriers. Notably, the final solid–solid transformation from Na_2_S_2_* to Na_2_S* is identified as the rate-determining step with the highest energy barrier across all three models. Nevertheless, pronounced differences in thermodynamic driving force are observed among the materials. The Gibbs free energy for this step in Na_0.25_TiO_2_ is −5.51 eV, which is substantially lower than that of Na_0.33_TiO_2_ (−3.67 eV) and TiO_2_ (−0.05 eV), demonstrating that Na_0.25_TiO_2_ exhibits optimal thermodynamic matching throughout the stepwise cleavage and conversion of NaPSs.

## Conclusions

In summary, an IEDC electrocatalyst is developed by engineering a continuously tunable electronic structure via electrochemical Na intercalation. By incorporating Na_0.25_TiO_2_ into the sulfur cathode, DPIs composed of S and Na_0.25_TiO_2_ are constructed for high-performance quasi-solid-state Na–S batteries. These DPIs offer a significantly larger catalytic active surface area than the conventional TPIs typically required for the SRR, thereby allowing a greater fraction of sulfur to participate in the conversion reactions. Furthermore, these interfaces exhibit high charge-transfer efficiency and significantly lower the activation energy and Gibbs free energy barriers associated with NaPS conversion. The results demonstrate that Na intercalation weakens Ti–O covalency by forming local Na–O–Ti configurations, activating surface oxygen atoms as electron-rich Lewis basic sites that improve the adsorption strength toward NaPSs. Concurrently, the intercalated Na acts as an electron donor, injecting electrons into the Ti 3d orbitals and reconstructing the potential energy surface, which substantially boosts both the ionic and electronic conductivity of TiO_2_. However, at high Na concentrations, excessively strong adsorption increases the energy barrier for NaPS conversion. Meanwhile, electron localization caused by the energy-level splitting between occupied and unoccupied states of the Ti 3d orbital, combined with enhanced local Na^+^ ion–ion repulsion, significantly weakens the intrinsic electronic and ionic transport of Na_*x*_TiO_2_. Na_*x*_TiO_2_ achieves an optimal synergy between the adsorption strength toward NaPSs and the intrinsic electronic/ionic transport properties when *x* reaches 0.25. Consequently, the Na_0.25_TiO_2_/S cathode with high sulfur content exhibits exceptional sulfur utilization, high reversible capacity, prolonged cycling life, and robust adaptability to high sulfur mass loading, and successfully enables the construction of high-energy-density pouch cells. This work overcomes the limitations of sulfur conversion reactions at TPIs and paves the way for the development of high-energy-density, low-cost, and durable solid-state Na–S batteries.

## Experimental Methods

### Synthetic Samples

#### Synthesis of the Polymer Electrolyte

In a typical synthesis process of the polymer electrolyte (PE), 2.0 g of poly­(vinylidene fluoride-*co*-hexafluoropropylene) (PVDF-HFP, Sigma-Aldrich) pellets was dissolved in acetone (25 mL) under vigorous stirring at 50 °C for 3 h. The resulting milky solution was then maintained at 50 °C for 1.5 h without stirring to eliminate air bubbles. Subsequently, the solution was cast onto a polypropylene (PP) film (Celgard 2400) using a doctor blade to fabricate a PP@PVDF-HFP composite polymer film. Then, the film was dried under vacuum at 100 °C overnight to ensure complete solvent removal. The dried composite polymer film was immersed in a 1 M NaPF_6_/diglyme electrolyte (DoDochem) for 20 h to obtain the final quasi-solid-state PE. The Na-ion conductivity of this PE is 1.5 mS cm^–1^ (at room temperature), which was tested by an impedance method.^63^ This PE possesses a thickness of ∼30 μm and contains 35 wt % 1 M NaPF_6_/diglyme.

#### Synthesis of Na_*x*_TiO_2_ Electrocatalysts

Commercial anatase titanium oxide (TiO_2_, 99.8% metals basis, 30 nm, Aladdin, 90 wt %) was mixed with a conductivity agent (acetylene black, AB, 10 wt %) and dispersed in *N*-methyl-2-pyrrolidone (NMP) to form a uniform slurry. The resulting slurry was sprayed onto carbon paper (CP, 5 cm × 8 cm) using an airbrush and then dried at 50 °C overnight under vacuum to yield the TiO_2_/C electrode, with 400–450 mg mass loading. The assembling process for pouch cells was carried out in an argon-filled glovebox (H_2_O < 0.1 ppm, O_2_ < 0.1 ppm). The pouch cells were assembled by pairing the TiO_2_/C electrode with a sodium metal anode and 1 M NaPF_6_/diglyme electrolyte within an aluminum plastic film, which then were discharged at 10 mA g^–1^ to specific capacities (time) to synthesize a series of Na_*x*_TiO_2_ (0 < *x* < 1) solid solution phases. Finally, the Na_*x*_TiO_2_ powders were recovered by scraping them from the CP, washed three times with deionized water and ethanol, and dried under vacuum at 60 °C overnight.

#### Preparation of the 0.2 M Na_2_S_6_ Solution

The 0.2 M Na_2_S_6_ solution was prepared by mixing sodium sulfide (Na_2_S, 95%, anhydrous, Aladdin) and sulfur powder (S, 99.98% trace metals basis, Sigma-Aldrich) in tetraethylene glycol dimethyl ether (TEGDME) at a molar ratio of 1:5. The mixture was stirred at 60 °C overnight. All the above operations were carried out in the glovebox.

#### Preparation of the 0.5 M Na_2_S_6_ Catholyte

The 0.5 M Na_2_S_6_ solution was prepared by mixing Na_2_S and S in a 1 M NaTFSI/TEGDME electrolyte (DoDochem) at a molar ratio of 1:5. The mixture was stirred at 60 °C overnight. All the above operations were carried out in the glovebox.

### Material Characterization

Powder X-ray diffraction (XRD) patterns were recorded on a Bruker D2 PHASER X-ray diffractometer using Cu Kα radiation (λ = 1.5418 Å) with an operating voltage of 30 kV and a current of 10 mA in the range of 10–80°. The morphology of the products and the surface morphologies of electrodes after cycling were examined by a scanning electron microscope (SEM, TESCAN VEGA3) with an energy-dispersive X-ray spectroscopy (EDS) detector. To investigate chemical states and bonding environments, X-ray photoelectron spectroscopy (XPS, Thermo Fisher ESCALAB 250Xi) was performed using Ar-ion sputtering. X-ray absorption spectra (XAS) for Ti *K*-edges and O *K*-edges were collected at the medium beamline (MEX-1) station of the Australian Synchrotron, part of ANSTO. The resulting X-ray absorption near-edge structure (XANES), extended X-ray absorption fine structure (EXAFS), and soft X-ray absorption structure (sXAS) data were processed by Athena and Artemis software. Ultraviolet–visible (UV–vis) absorption spectra were recorded using a JASCO V-670 UV–vis–NIR spectrometer equipped with an integrating sphere. Raman spectra were acquired using a Horiba LabRAM HR Evolution system with a 532 nm excitation laser.

### Visualized Polysulfide (Na_2_S_6_) Adsorption Test

The as-prepared 0.2 M Na_2_S_6_ solution was diluted to the desired concentration for the adsorption tests. Subsequently, 15 mg of Na_*x*_TiO_2_ (*x* = 0, 0.17, 0.25, 0.33, and 0.5) was dispersed into 3.5 mL of the diluted Na_2_S_6_ solution, which was left undisturbed for several hours to allow for sedimentation and complete adsorption. All of the above operations were carried out in the glovebox.

### Na_2_S Precipitation Test

Na_*x*_TiO_2_ (*x* = 0, 0.17, 0.25, 0.33, and 0.5), Ketjen Black (KB, ECP-600JD), and PVDF were dispersed in NMP solvent in a ratio of 8:1:1 to obtain a uniform slurry, which was subsequently cast on CP by a doctor blade and vacuum-dried at 50 °C for overnight, to obtain the Na_*x*_TiO_2_/CP electrodes (φ12 mm, 1.5 mg cm^–2^). The precipitation experiments of Na_2_S were investigated by CR2032-type coin cells on an electrochemical workstation (CHI660E, CH Instruments). The Na_*x*_TiO_2_/CP electrode was applied as a current collector for 0.2 M Na_2_S_6_ solution. Twenty-five μL of Na_2_S_6_ solution was added dropwise onto the Na_*x*_TiO_2_/CP, while 80 μL of the 1 M NaTFSI/TEGDME electrolyte was added to the Na anode. The assembled cell was first discharged to 1.3 V and then kept at a potentiostatic voltage of 1.25 V until the current was below 10^–5^ A.

### Symmetric Cyclic Voltammetry (CV) Test

Two identical Na_*x*_TiO_2_/CP electrodes were used to assemble a symmetrical CR2032 coin-type cell with 50 μL of the 0.5 M Na_2_S_6_ catholyte. The cell was tested at an electrochemical window of −1 ∼1 V with a sweep rate of 200 mV s^–1^.

### Electrochemical Measurements

Commercial sulfur powder was processed via high-energy ball milling for 1 h to produce micropowder sulfur (mp-S) with a particle size distribution of 20–40 μm. To prepare the cathode, mp-S (70 wt %) was mixed with a conductivity agent (Ketjen Black, KB, ECP-600JD, 10 wt %), a binder (PVDF-HFP, 10 wt %), and the as-prepared Na_*x*_TiO_2_ electrocatalysts (10 wt %). This mixture was dispersed in NMP to form a homogeneous slurry. The slurry was cast onto carbon-coated aluminum foil using a doctor blade and dried under vacuum at 55 °C overnight, finally obtaining Na_*x*_TiO_2_/S cathodes with a sulfur mass loading of 1.5–3.0 mg cm^–2^. Unless otherwise specified for the investigation of mass loading effects on electrochemical performance, a standard sulfur mass loading of 1.5 mg cm^–2^ was employed for all cells. Quasi-solid-state Na–S batteries were assembled in CR2023 coin cells in a glovebox, which were made up of a Na_*x*_TiO_2_/S cathode (φ12 mm), one piece of PE (φ16 mm), and a Na metal disk (φ12 mm). Galvanostatic discharge/charge tests of the batteries were carried out on a battery test system (NEWARE BTS, Neware Technology Co., Ltd., China) in the range of 1.0–2.8 V (vs Na^+^/Na) at room temperature (25 °C). CV, symmetric CV, and electrochemical impedance spectroscopy (EIS) were carried out by CHI660E. CV was performed at 0.2–0.8 mV s^–1^ under an electrochemical window of 1.0–2.8 V. The frequency range of the EIS test was 0.1 MHz to 10 mHz.

### DFT Calculations

First-principles calculations were performed using the Vienna Ab-initio Simulation Package (VASP).^64,65^ The projector augmented wave (PAW) pseudopotentials were employed to treat the electron–ion interactions,^66^ and the generalized gradient approximation (GGA) parametrized by Perdew–Burke–Ernzerhof (PBE) was used for the electron–electron exchange-correction functional.^67^ The cutoff kinetic energy for plane waves was set to 400 eV. Geometric and structural optimizations were carried out with a force convergence threshold of 0.02 eV/Å and an energy convergence criterion of 10^–6^ eV. Brillouin zone sampling was conducted using a 3 × 4 × 3 *k*-point mesh within the Monkhorst–Pack scheme,^68^ for a supercell model of Na_*x*_TiO_2_ containing 24 formula units. All the structures were relaxed with a convergence tolerance of 1 × 10^–4^ eV. Note that the selected dynamic method was adopted to relax the Na atoms in the Na_*x*_TiO_2_ substrate, while only Na_2_S_*n*_ and the first three layers of the substrate were relaxed in the absorption model. The *k*-point grid with a separation of 0.05 Å^–1^ centered at the Γ-point was automatically set for the structural relaxation. A much denser *k*-point grid of 0.04 Å^–1^ was set for static calculations. The convergence criterion for the electronic self-consistency loop was set to 1 × 10^–4^ eV. To appropriately describe the electronic states of Ti 3d-states, the *U*_eff_ = *U* – *J* value was set as 5.8 eV for DOS (Density of States) calculations.^69^ The Na diffusion energy barriers were calculated based on the climbing image nudged elastic band (CI-NEB) method.^70^ In the CI-NEB calculations, the initial and final structures were fully relaxed, and six intermediate structures in between were linearly interpolated. To evaluate the strength of bonding between Na_2_S_*n*_ molecules and the Na-doped TiO_2_ substrate, we calculated the binding energy using the following equation: *E*_b_ = (*E*_sub._ + *E*_Na_2_S_*n*__) – *E*_sub.+Na_2_S_*n*__, where *E*_sub._ and *E*_Na_2_S_n__ are the total energy of the Na_*x*_TiO_2_ substrate and the isolated Na_2_S_*n*_ molecule, respectively. E_sub.+Na_2_S_*n*__ represents the total energy of the Na-doped TiO_2_ substrate after absorbing the Na_2_S_*x*_ molecule. The Gibbs free energy change (Δ*G*) of each step was calculated using the formula: Δ*G* = Δ*E* + ΔZPE – TΔS, where Δ*E* is the electronic energy difference directly obtained from DFT calculations, ΔZPE is the zero-point energy difference, *T* is the room temperature (298.15 K), and Δ*S* is the entropy change.

## Supplementary Material



## Notes

Additional simulated TiO_2_ crystal lattice structure, XRD, EDS, charge analysis, Ti *K*-edge XANES and FT-EXAFS, static Na_2_S_6_ adsorption experiment, symmetrical CV, Tafel slope, electrochemical performance, XPS, SEM, simulated migration path of Na^+^ ions on the TiO_2_ surface, tables for calculation of *x* content in Na_*x*_TiO_2_ design, parameters of pouch cells, and comparison of electrochemical performance with the previous reports (PDF)
