# Supplementary material for: Expanding Catalytic Active Surface via Dual-Phase Interfaces for High-Energy-Density Quasi-Solid-State Na–S Batteries
Source: ACS Nano. 2026 Sep 2;20(36):25124–37. doi: 10.1021/acsnano.6c12130 (PMC13585175; doi:10.1021/acsnano.6c12130)
Supplement: Supplementary file 1 [file nn6c12130_si_001.pdf]

Supporting Information

Expanding Catalytic Active Surface via Dual-Phase Interfaces for High-Energy-Density Quasi-Solid-State Na-S Batteries

Xuan Lu,[a,b,‡,*] Sicong Jiang,[c,‡] Hongyang Zhao,[a,‡] Xiaobo Zheng,[d,‡] Yiwen Zhang,[b] Kesong Yang,[c,*] Shujiang Ding,[a,*] Xiaofei Hu,[a,b,*] Weiyang Li[b,*]

[a] School of Chemistry, Engineering Research Center of Energy Storage Materials and Devices (Ministry of Education), Xi'an Key Laboratory of Sustainable Energy Materials Chemistry, Xi'an Jiaotong University, Xi'an, Shaanxi, 710049, People's Republic of China

[b] Thayer School of Engineering, Dartmouth College, 14 Engineering Drive, Hanover, New Hampshire 03755, United States

[c] Aiiso Yufeng Li Family Department of Chemical and NanoEngineering, University of California San Diego, 9500 Gilman Drive, La Jolla, California 92093-0448, United States

[d] Centre for Clean Energy Technology, School of Mathematical and Physical Sciences, Faculty of Science, University of Technology Sydney, Sydney, NSW 2007, Australia

[‡] X. L., S. J., H. Z. and X. Z. contributed equally to this work.

*Address correspondence to: Xuan Lu (E-mail: xuan.lu@xjtu.edu.cn)

Kesong Yang (E-mail: kesong@ucsd.edu)

Shujiang Ding (E-mail: dingsj@mail.xjtu.edu.cn)

Xiaofei Hu (E-mail: xiaofei.hu@xjtu.edu.cn)

Weiyang Li (E-mail: weiyang.li@dartmouth.edu)

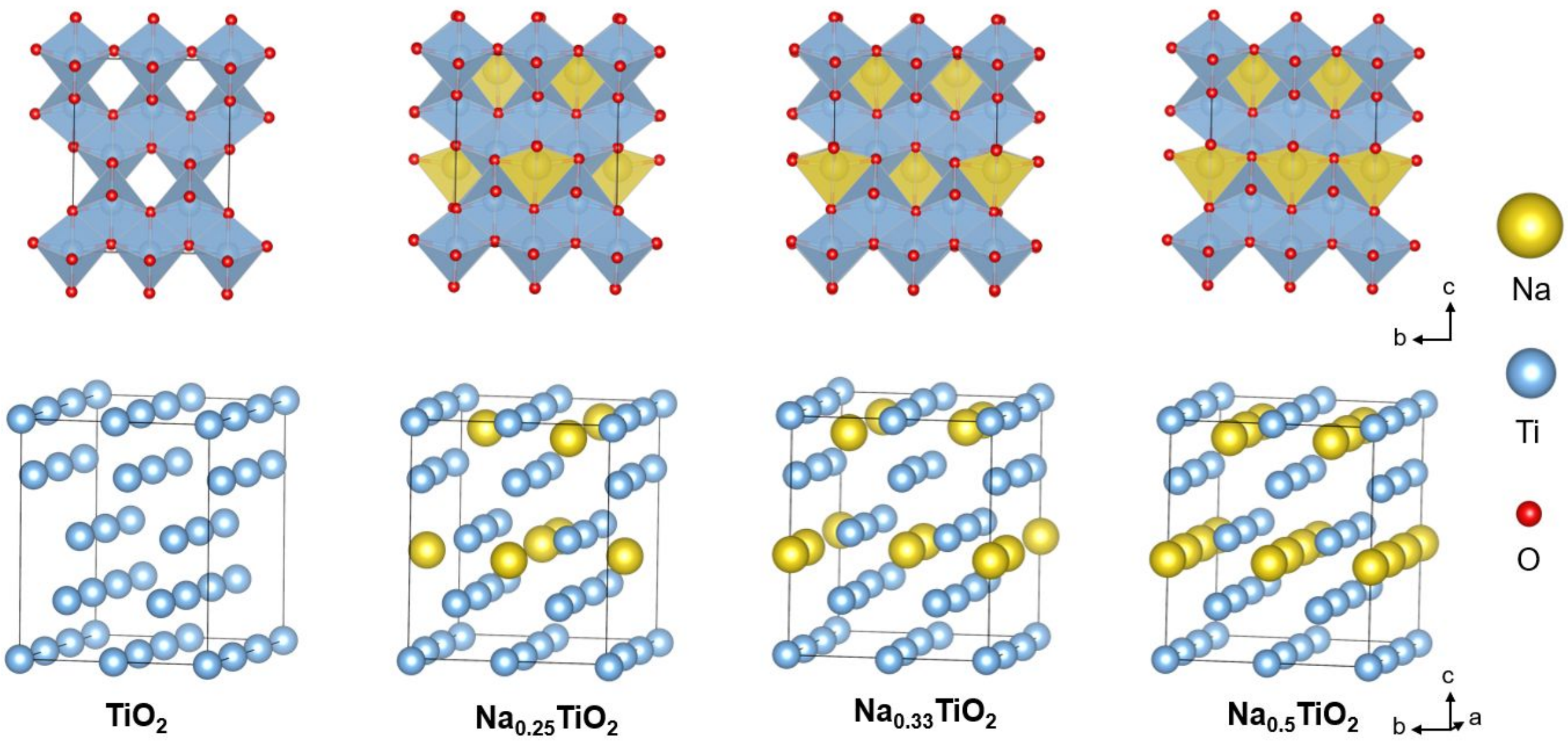

Figure S1. Schematic illustration of the structural evolution of TiO_2 upon Na^+ intercalation. Top: Projection views of TiO_2, $Na_{0.25}TiO_2$, $Na_{0.33}TiO_2$, and $Na_{0.5}TiO_2$ structures along the [010] direction, showing the increasing occupancy of Na^+ ions (yellow) in the TiO_2 lattice. Bottom: Corresponding 3D structural models highlighting the positions of Ti (blue) and Na (yellow) atoms.

The lattice parameters of TiO_2 exhibit a progressive expansion with increasing Na intercalation (x). Specifically, the a-axis increases from 3.899 Å at $x = 0.00$ to 3.938 Å at $x = 0.50$, while the b-axis undergoes a more pronounced expansion from 3.899 Å to 4.162 Å. The c-axis also shows a moderate increase from 9.646 Å to 9.933 Å. Despite these variations, the overall lattice expansion remains relatively minor, suggesting that the TiO_2 host framework accommodates Na^+ intercalation with minimal structural disruption and maintains good phase stability.

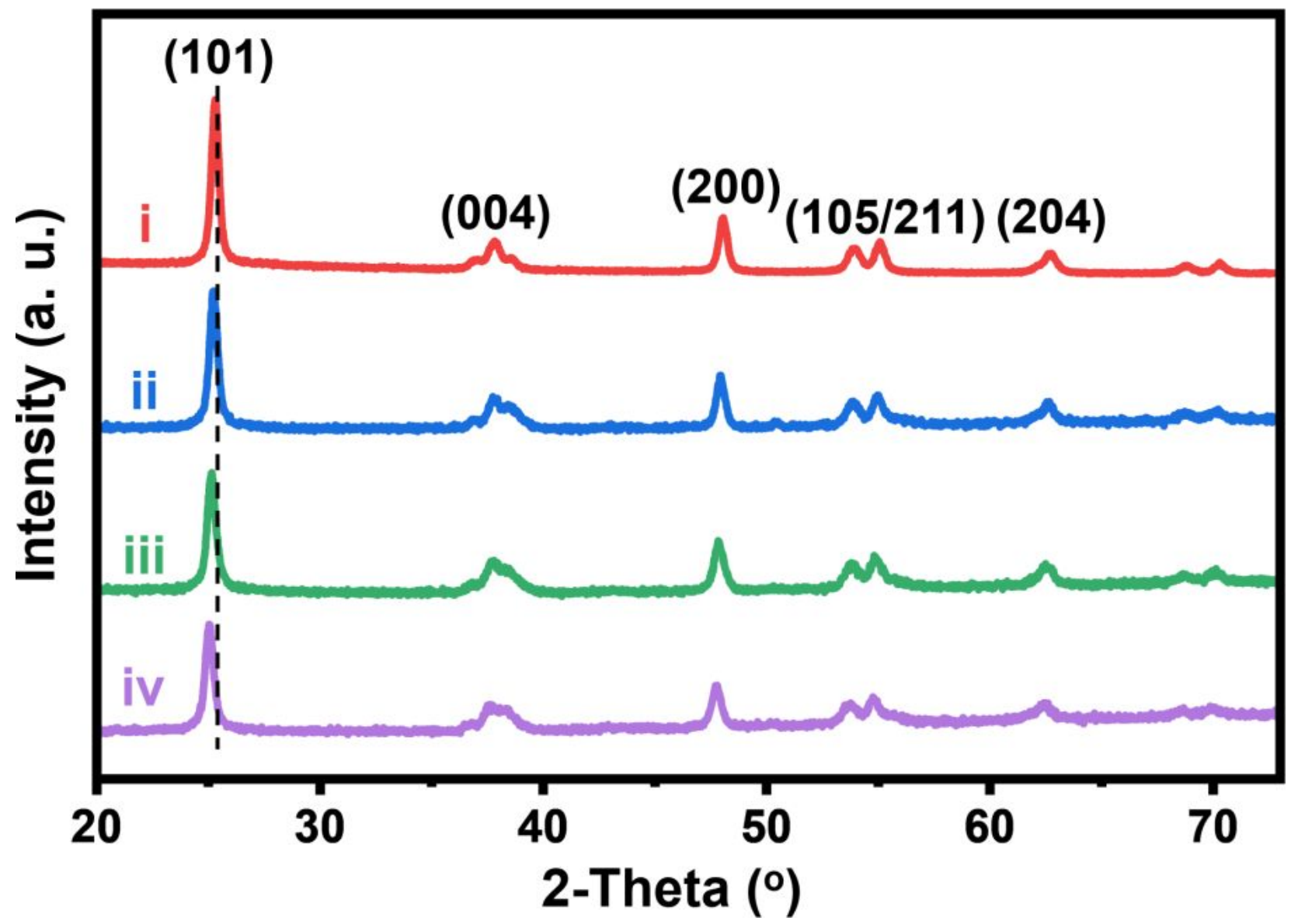

Figure S2. XRD patterns of pristine TiO_2 (i) and Na_xTiO_2 (ii-iv).

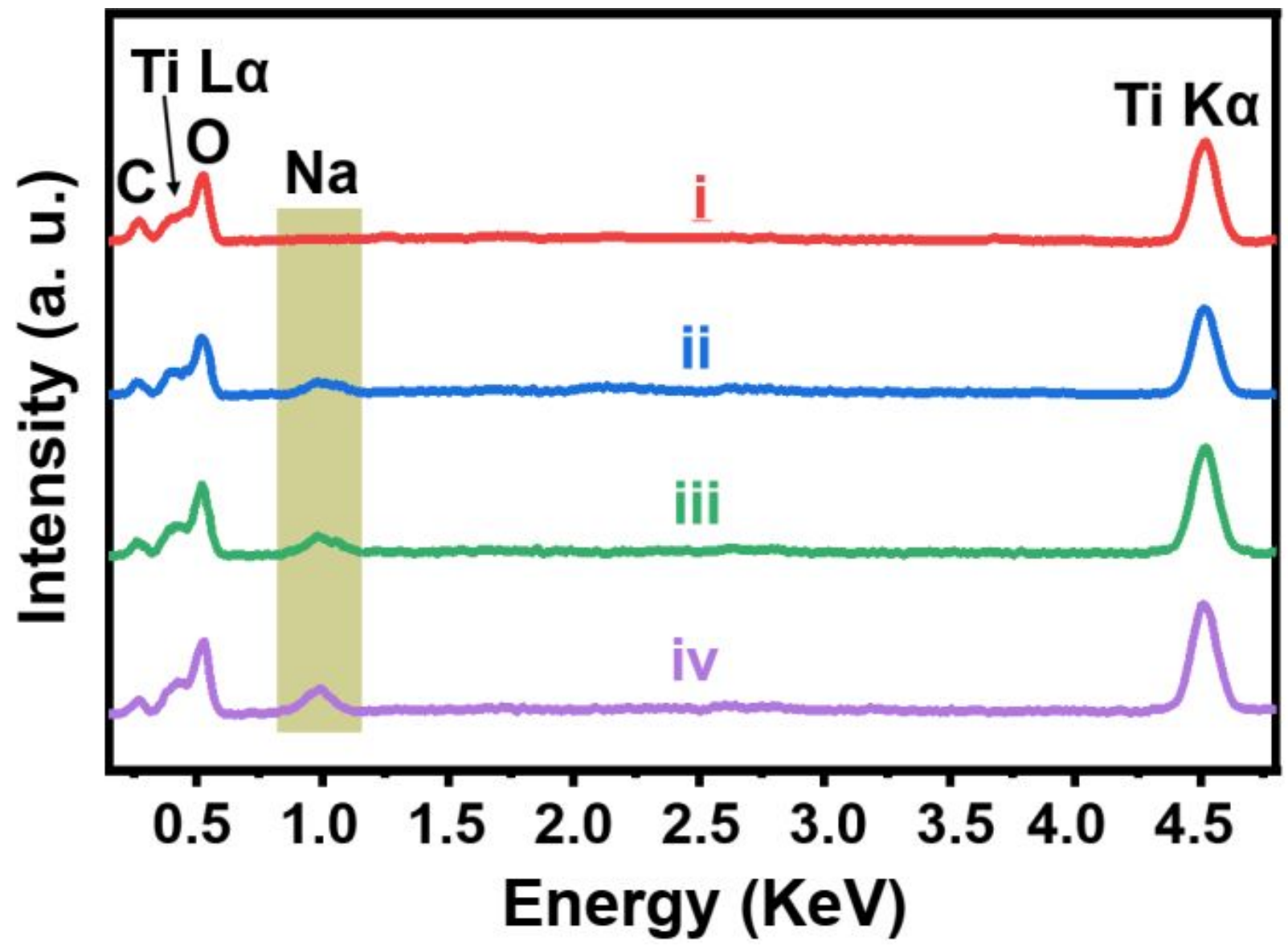

Figure S3. EDS spectra of pristine TiO_2 (i) and Na_xTiO_2 (ii-iv).

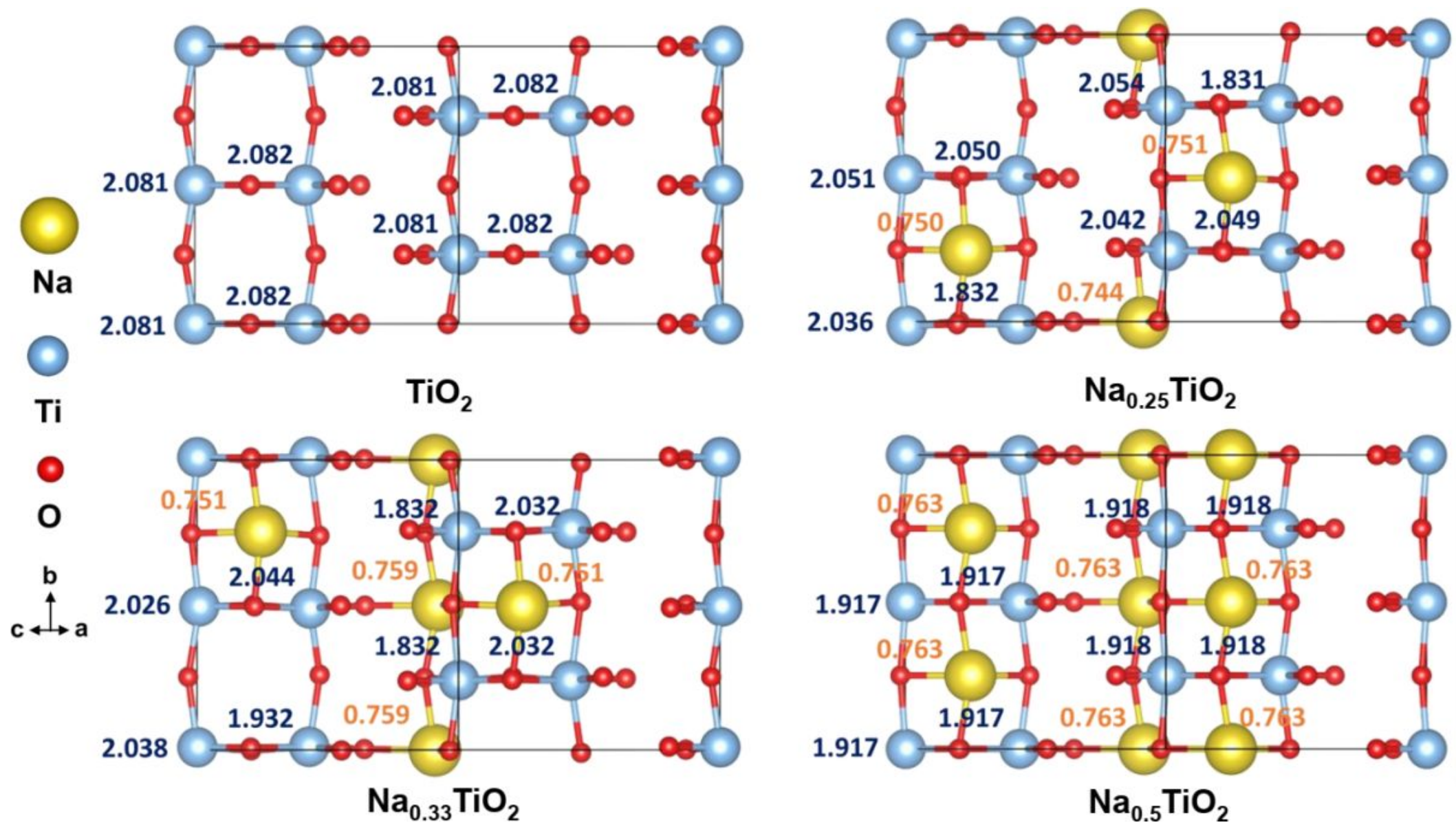

Figure S4. Charge density distribution on the (101) crystal facet of Na_xTiO_2.

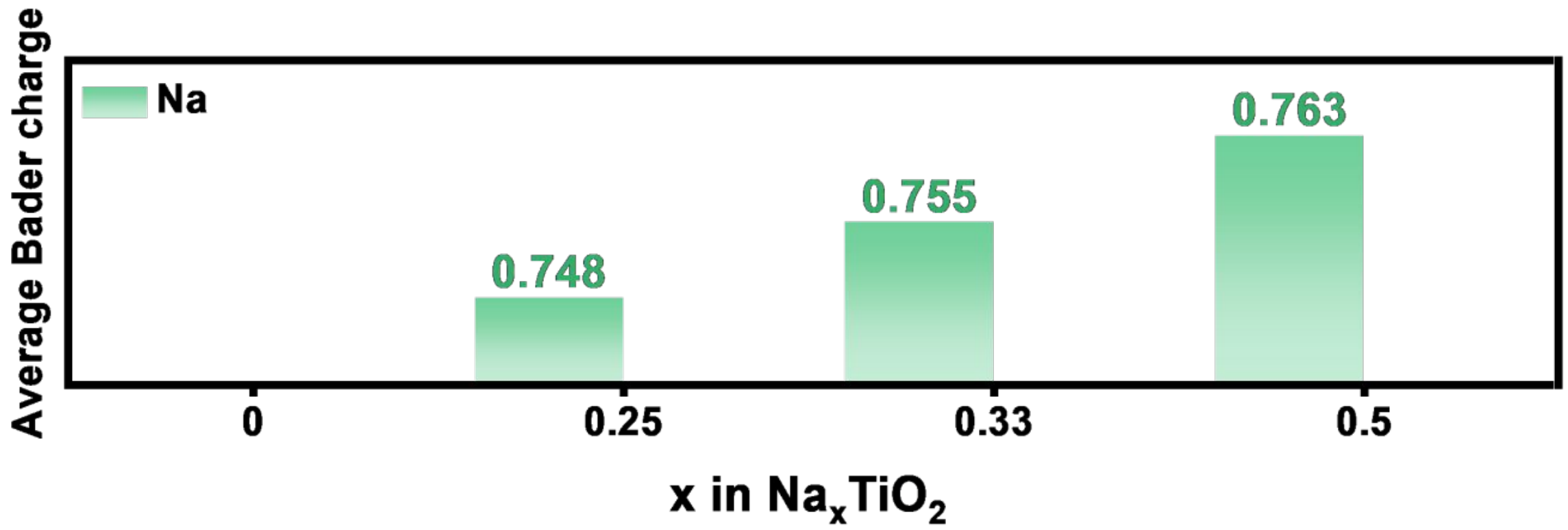

Figure S5. Charge variations of Na atom based on Bader charge analysis.

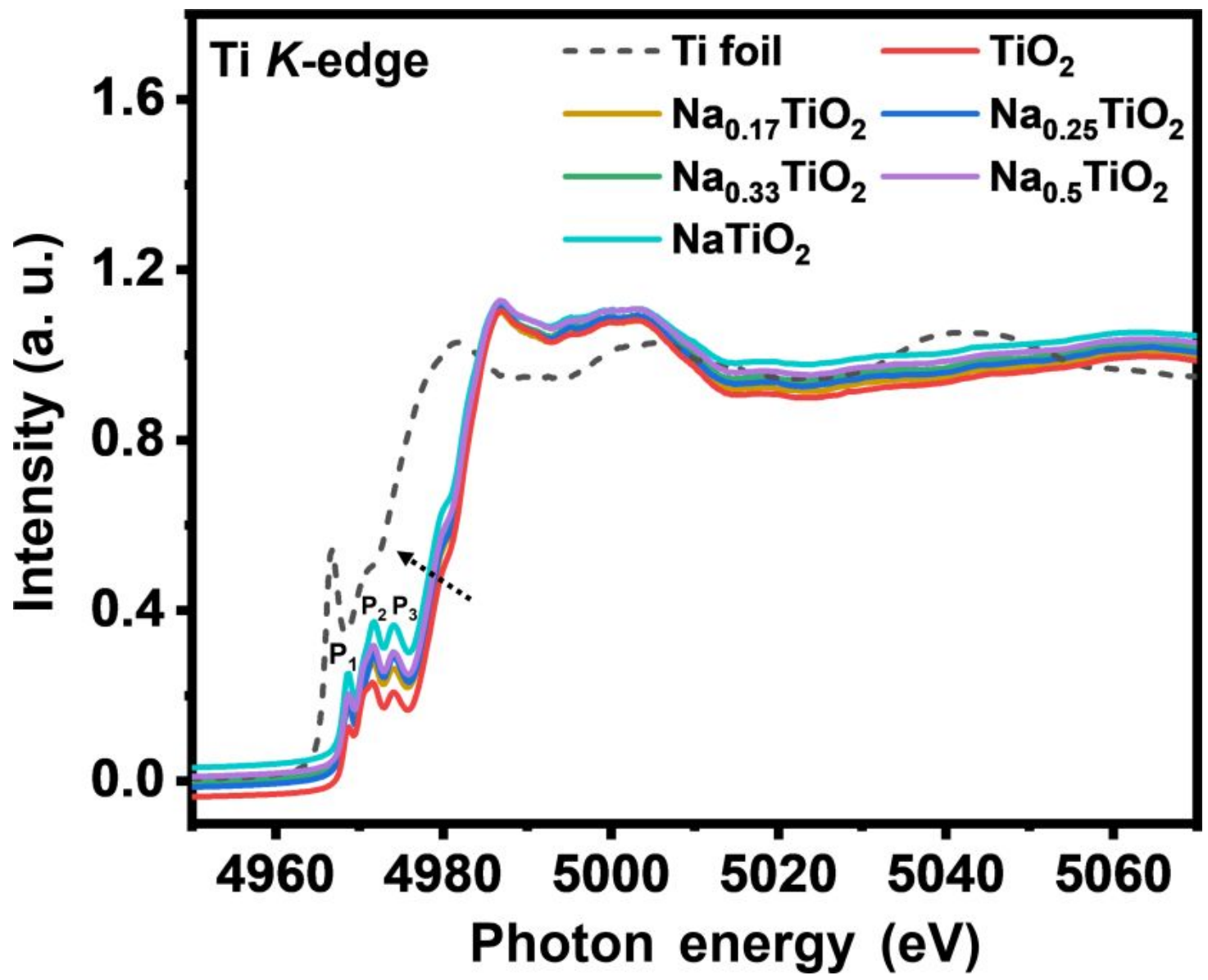

Figure S6. Ti *K*-edge XANES spectra of pristine TiO_2 and Na_xTiO_2.

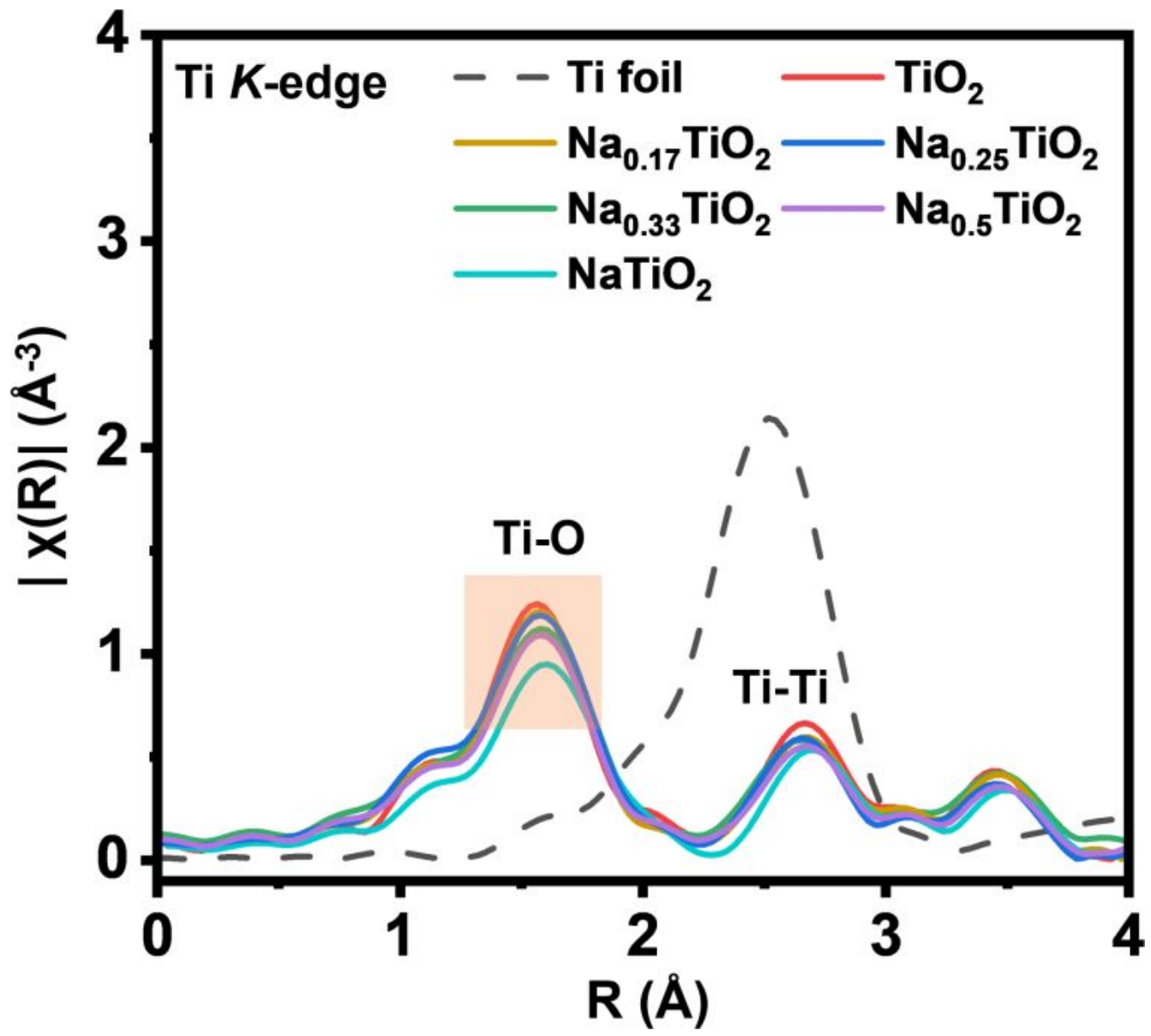

Figure S7. Ti *K*-edge FT-EXAFS spectra of pristine TiO_2 and Na_xTiO_2.

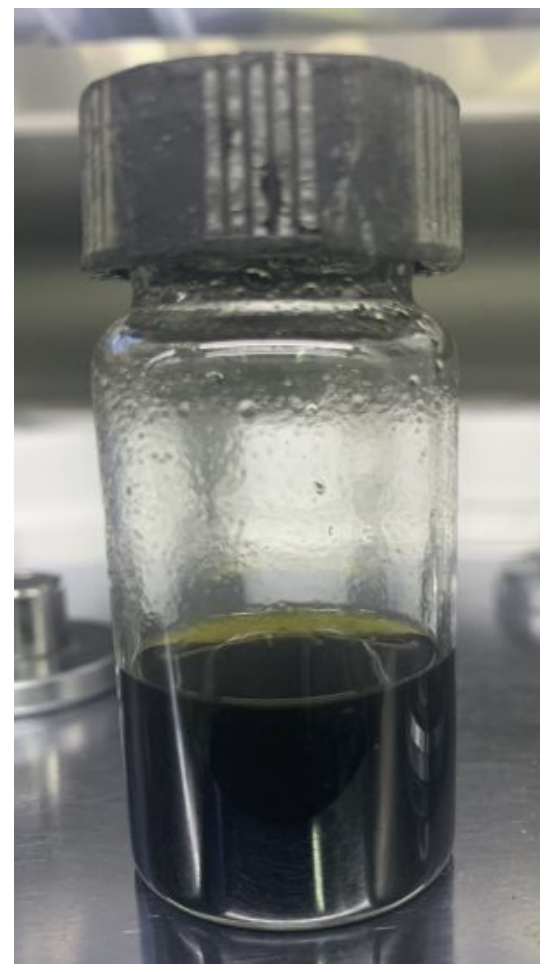

Figure S8. Digital photograph of static Na_2S_6 adsorption experiment using carbon (acetylene black, AB) as an adsorbent.

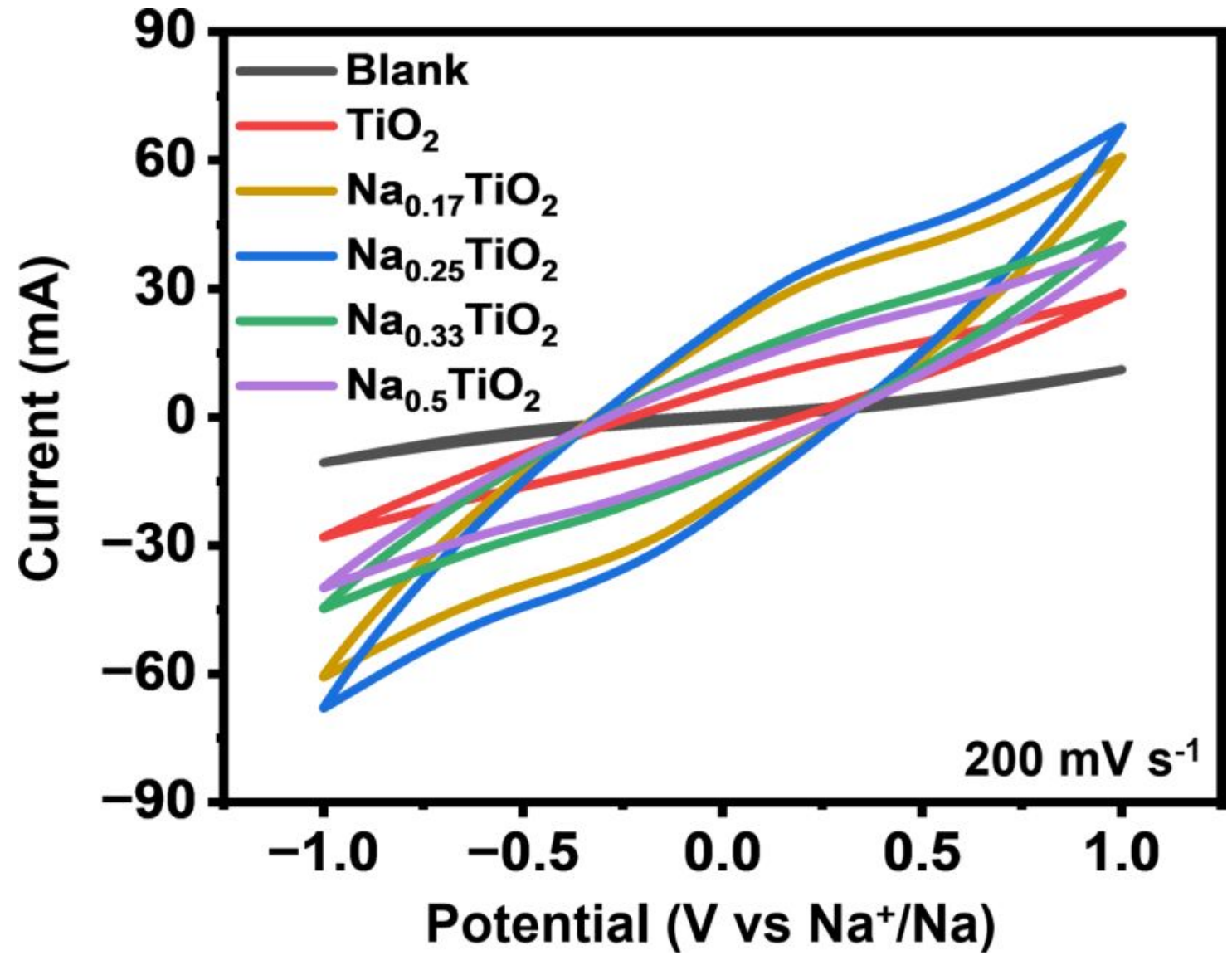

Figure S9. Symmetrical CV curves of TiO_2 and Na_xTiO_2 at 200 mV s^{-1}.

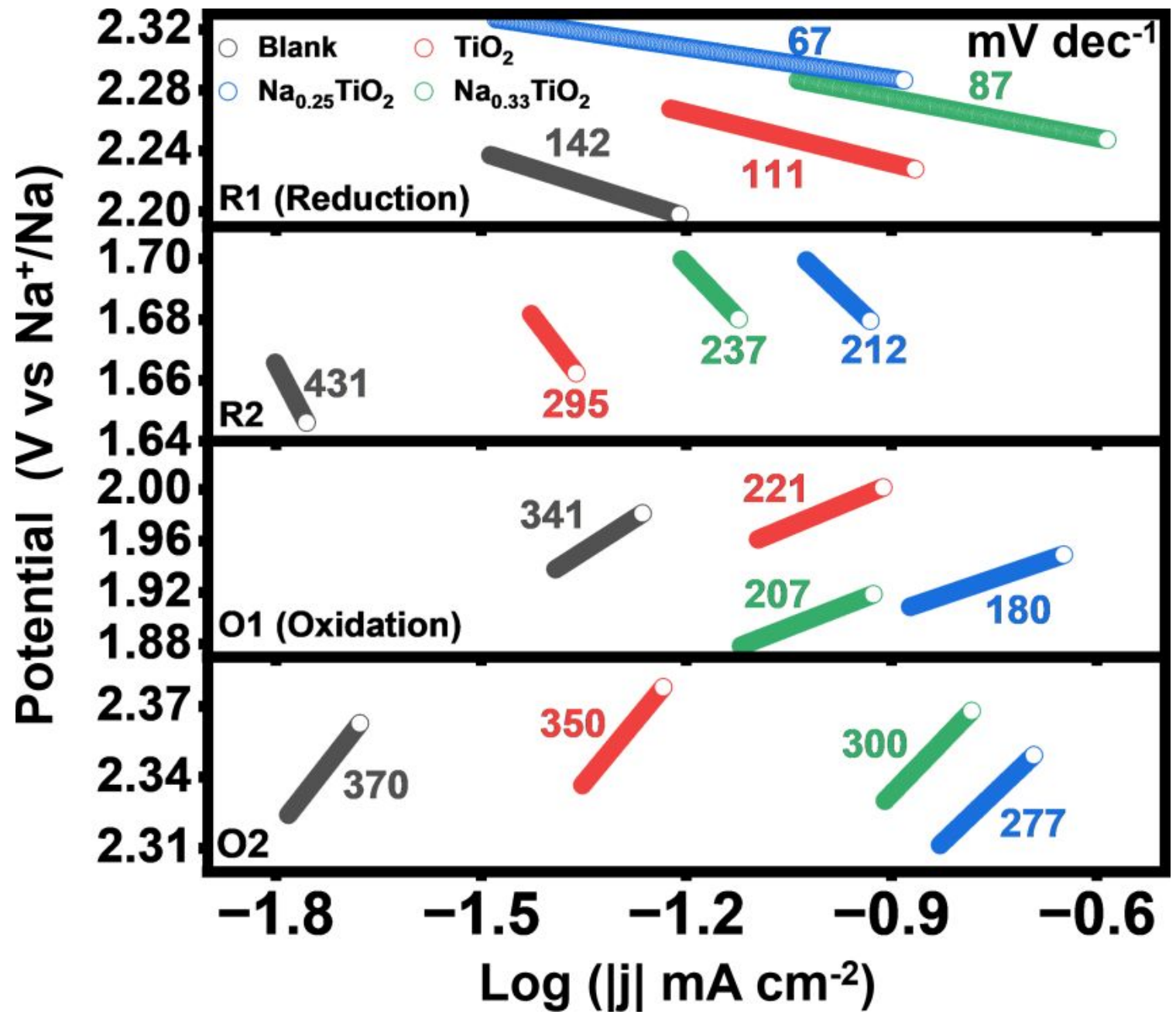

Figure S10. Tafel slopes of various cathodes utilizing different electrocatalysts.

$Na_{0.25}TiO_2$/S cathode exhibits the smallest Tafel slopes for both R_1 and R_2, with values of 67 and 212 mV dec^{-1}, respectively, indicating that precise electronic structure modulation of TiO_2 plays a critical role in facilitating the $S_8 \rightarrow Na_2S$ conversion. A consistent trend is observed for the O_1/O_2 peaks, where the $Na_{0.25}TiO_2$/S cathode shows the smallest slopes (180/277 mV dec^{-1}). In contrast, the slopes for $Na_{0.33}TiO_2$/S, TiO_2/S, and the blank/S cathode increase to higher values of 207/300, 221/350, and 341/370 mV dec^{-1}, respectively, revealing the superior capability of $Na_{0.25}TiO_2$ in promoting the S oxidation reaction from Na_2S back to S_8.

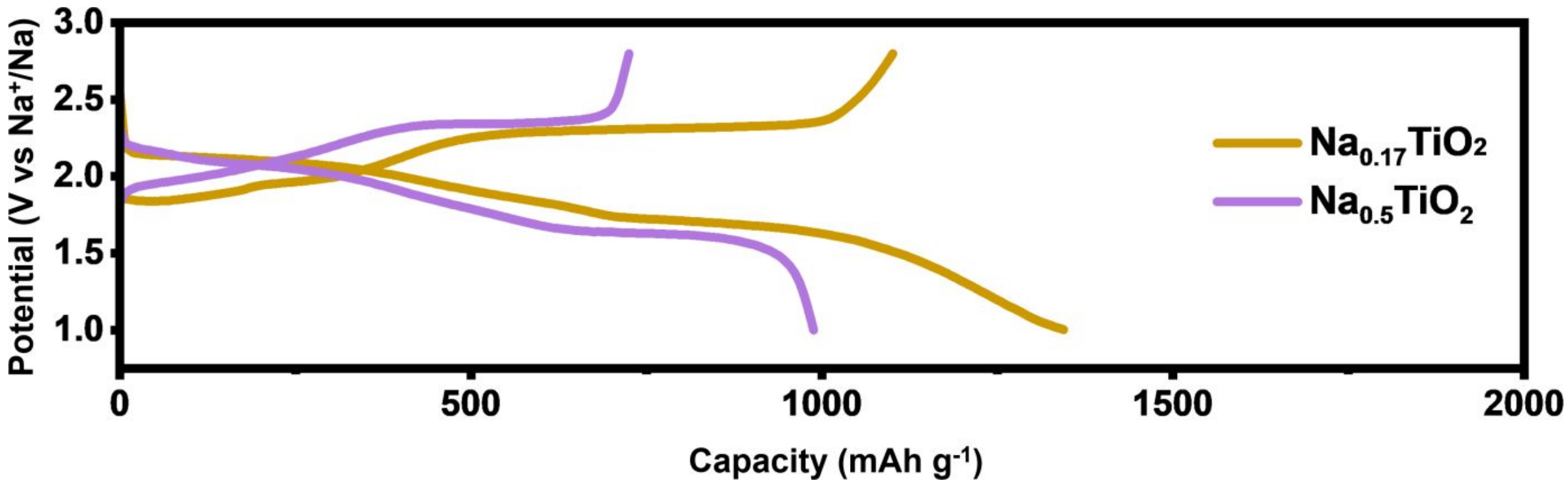

Figure S11. Galvanostatic discharge-charge profiles of $Na_{0.17}TiO_2$/S and $Na_{0.5}TiO_2$/S cathodes at 0.1C.

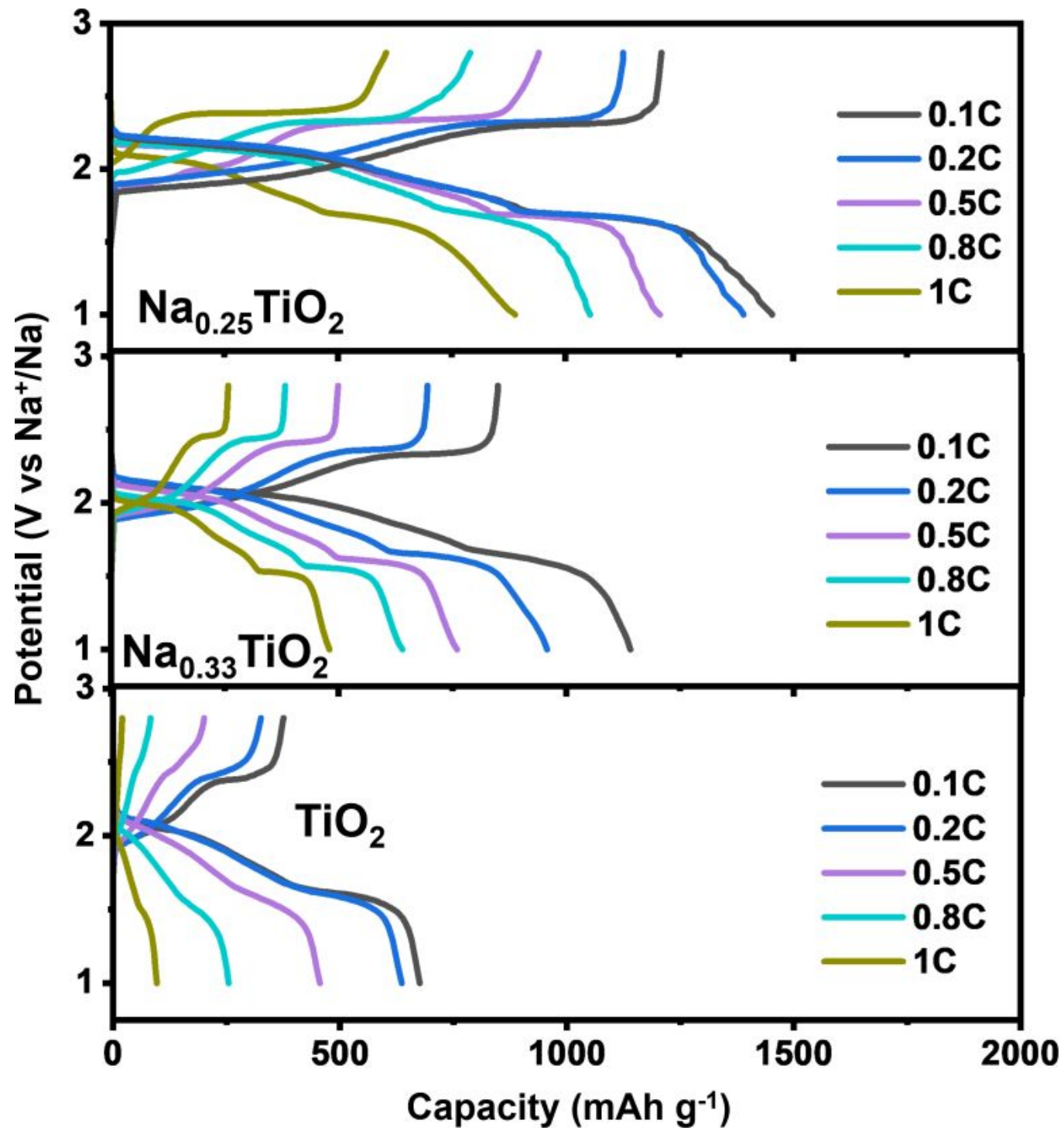

Figure S12. Rate performance of cells with Na_xTiO_2 electrocatalysts at 0.1-1C.

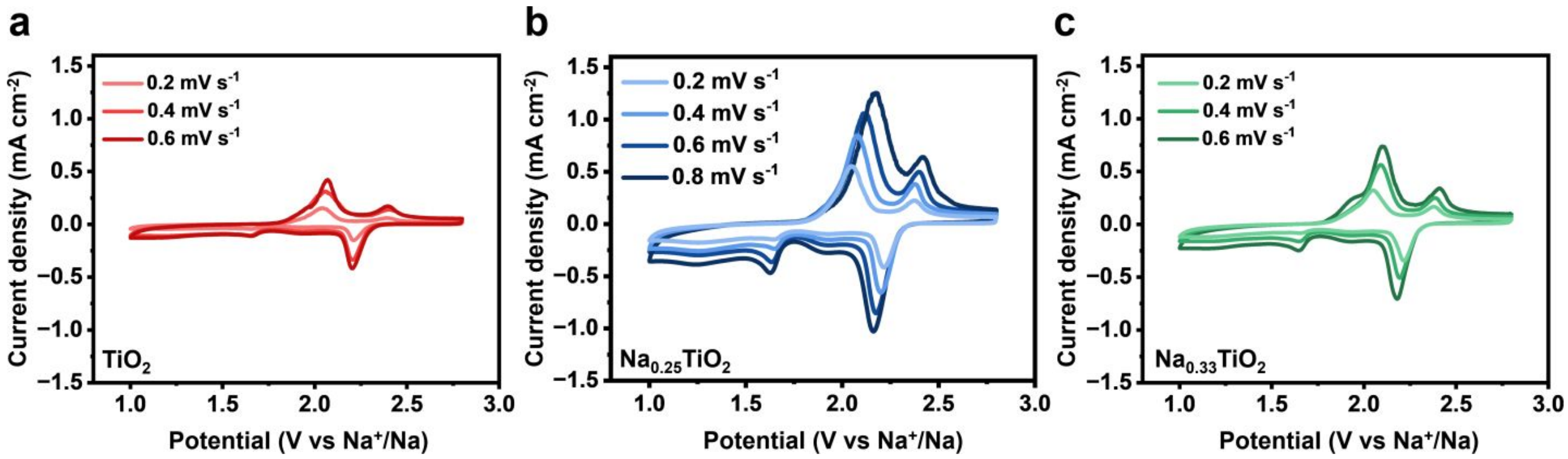

Figure S13. (a-c) CV curves at various scan rates of TiO_2 (a), $Na_{0.25}TiO_2$ (b), and $Na_{0.33}TiO_2$ (c).

`

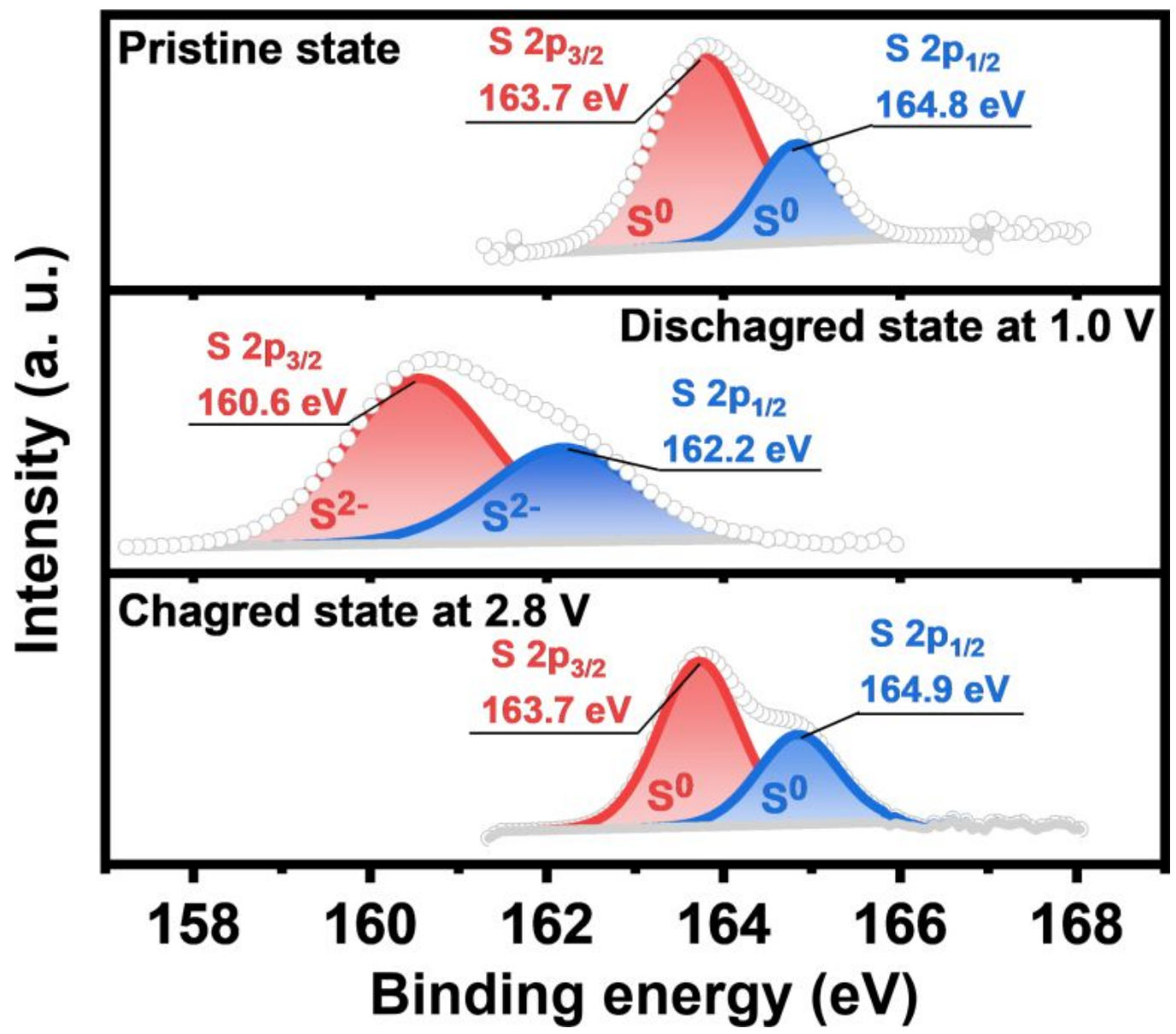

Figure S14. High-resolution S 2p XPS spectra of $Na_{0.25}TiO_2$/S cathode at different cycling states.

The pristine $Na_{0.25}TiO_2$/S cathode exhibits two characteristic S 2p peaks at 163.7 and 164.8 eV, which are assigned to the S $2p_{3/2}$ and S $2p_{1/2}$ components of elemental sulfur (S^0), respectively.[1,2] Upon discharging to 1.0 V, the characteristic S^0 peaks disappear, while two new peaks emerge at 160.6 and 162.2 eV, corresponding to the S $2p_{3/2}$ and S $2p_{1/2}$ components of S^{2-} in Na_2S.[3-5] Upon subsequent charging to 2.8 V, the S^0-related peaks are fully restored, whereas the Na_2S-associated peaks vanish.

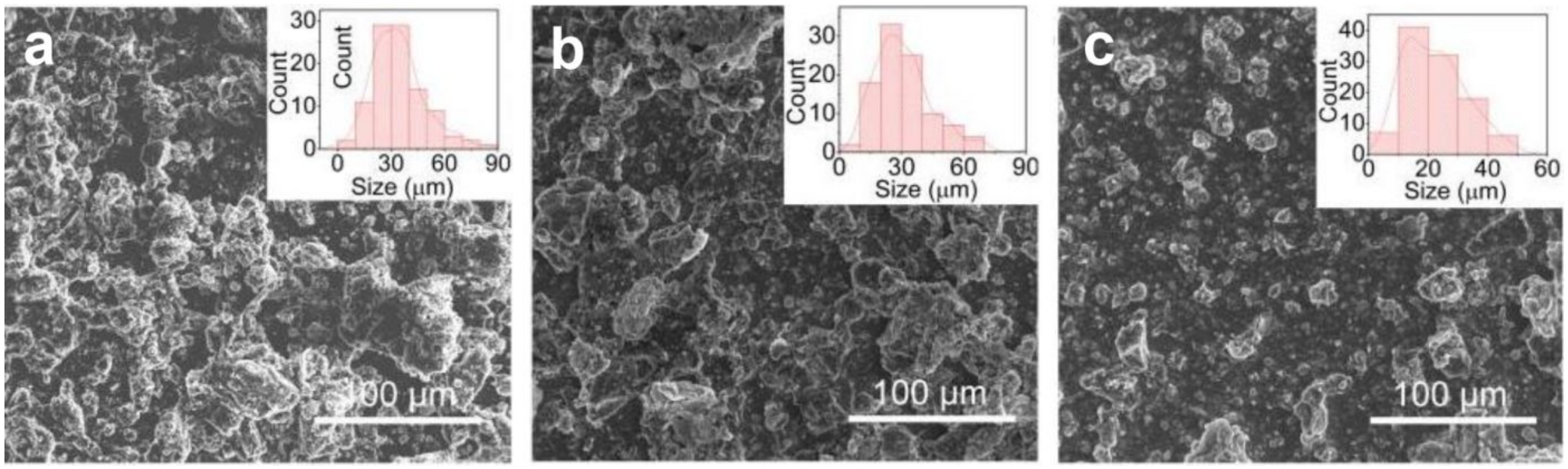

Figure S15. (a-c) Morphological evolution of the $Na_{0.25}TiO_2$/S cathode at different cycling stages: initial state (a), after 50 cycles (b), and after 100 cycles (c).

Specifically, the S particles, which initially range from 20 to 40 μm (**Figure S15a**), gradually decrease to 20-30 μm after 50 cycles (**Figure S15b**), and further refine to 10-20 μm by the 100th cycle (**Figure S15c**). This unique structural evolution is attributable to the superior catalytic activity of $Na_{0.25}TiO_2$: i) the abundant catalytically active sites on the $Na_{0.25}TiO_2$ surface effectively lower the nucleation barrier for Na_2S formation, inducing the homogeneous nucleation and uniform deposition of Na_2S at a reduced scale, rather than the localized overgrowth and disordered agglomeration typically observed in kinetically sluggish systems; ii) the electrocatalyst accelerates the reaction kinetics at the active material/ion/electron triple-phase interfaces. This ensures that large S particles are fully utilized during redox processes, facilitating their *in-situ* reconstruction into more electrochemically active micro-/nanostructures. As a result, this catalyst-induced grain-refinement effect substantially increases the specific surface area of the active material, exposes more accessible reaction sites, and shortens Na^+/e^- transport pathways, thereby underpinning the high reversible capacity and long-term cycling stability of the battery.

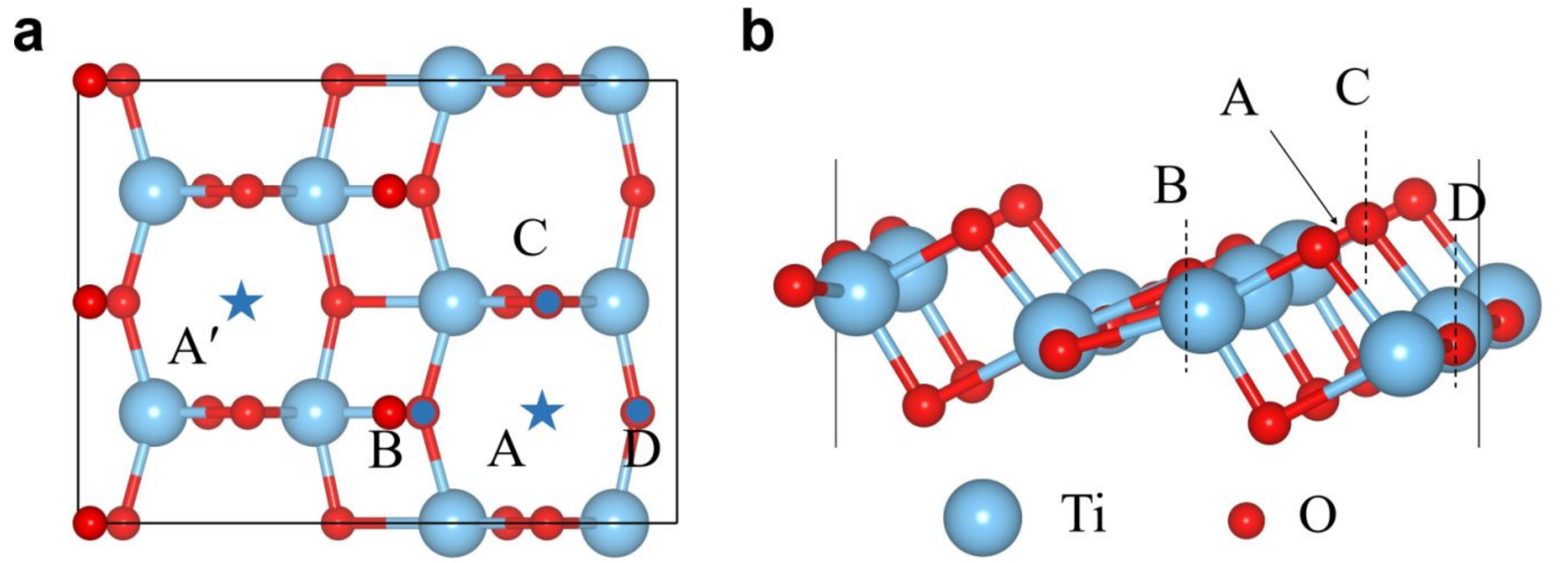

Figure S16. (a) Top and (b) side views of A-B-C-D four possible Na adsorption sites on the pristine TiO_2 substrate. The star symbol represents the most stable position.

The results indicate that site A is the most energetically stable. Na atoms positioned at sites B and D spontaneously migrate to site A during structural relaxation, confirming their thermodynamic instability. In contrast, site C exists as a metastable state, though its energy remains significantly higher than that of site A. For instance, the energy difference between sites A and C in the $Na_{0.17}TiO_2$ model is 0.24 eV. Consequently, it is reasonable to expect that surface Na^+ diffusion occurs primarily between adjacent hollow sites (A → A′), rather than through transitions between different types of adsorption sites.

Table S1. Relationship between Ti^{3+}/Ti^{4+} ratio and Na content x in Na_xTiO_2.

Ti^{3+}/Ti^{4+} Ratio (R)	Ti^{4+} (y)	Ti^{3+} (z)	Na Content ($x = z$)
0.33	0.75	0.25	0.25
0.5	0.67	0.33	0.33
1	0.5	0.5	0.5
∞ (all Ti^{3+})	0	1	1

To calculate the amount of Na^+ inserted into TiO_2 (i.e., the value of x in Na_xTiO_2), we begin by assuming there is 1 mole of Ti per mole of TiO_2. Let y represent the amount of Ti^{4+} and z the amount of Ti^{3+}. Given that the total Ti is conserved, we have $y + z = 1$. If the Ti^{3+}/Ti^{4+} ratio is known and denoted as R, then $z = R \cdot y$. Substituting into the first equation gives $y + R \cdot y = 1$, leading to $y = 1 / (1 + R)$, and therefore $z = R / (1 + R)$. Since each Ti^{4+} reduced to Ti^{3+} corresponds to the insertion of one Na^+ (which donates one electron), the amount of inserted Na is equal to z. Thus, the Na content $x = R / (1 + R)$. For example, if the Ti^{3+}/Ti^{4+} ratio is 0.33, then $x = 0.3 / (1 + 0.3) = 0.25$, and the resulting composition is $Na_{0.25}TiO_2$. This relationship is summarized in **Table S1**.

Table S2. Comparison of the electrochemical performance with previously reported quasi-solid-state Na-S batteries using polymer electrolyte.

Cathode	S content & S mass loading	Initial capacity at current density	S utilization	Reversible capacity after cycle number	Rate capability	Areal capacity at high S mass loading	Ref.
**$Na_{0.25}TiO_2/S$**	**70%, 1.5 mg cm^{-2}**	**1391.2 mAh g^{-1} at 0.2C**	**83.1%**	**800.3 mAh g^{-1} after 500 cycles**	**1C, 889.4 mAh g^{-1}**	**3.7 mAh cm^{-2} at 3mg cm^{-2}**	**This work**
poly(S-PETEA)@C	47%, 1.2-1.5 mg cm^{-2}	877 mAh g^{-1} at 0.1C	52.4%	736 mAh g^{-1} after 100 cycles	1C, 495 mAh g^{-1}	N/A	6
S-Co/NC-CF	N/A, N/A	~1300 mAh g^{-1} at 0.1C	77.6%	726 mAh g^{-1} after 100 cycles	1C, 183 mAh g^{-1}	N/A	7
SPAN	N/A, N/A	402.2 mAh g^{-1} at 0.12C	24%	327.4 mAh g^{-1} after 200 cycles	0.6C, 259.1 mAh g^{-1}	N/A	8
CNF/S	N/A, 2.0 mg cm^{-2}	~650 mAh g^{-1} at 0.2C	41.8%	550 mAh g^{-1} after 100 cycles	0.33C, ~600 mAh g^{-1}	N/A	9
S-CNT	60%, N/A	~775 mAh g^{-1} at 0.05C	46.3%	~380 mAh g^{-1} after 40 cycles	N/A	N/A	10
Comm-S	N/A, 1.2 mg cm^{-2}	713 mAh g^{-1} at 0.2C	42.6%	700 mAh g^{-1} after 200 cycles	1C, 600 mAh g^{-1}	N/A	11
S	70%, 1.2 mg cm^{-2}	~700 mAh g^{-1} at 0.2C	41.8%	~400 mAh g^{-1} after 200 cycles	N/A	N/A	12

Table S3. Design parameters of the quasi-solid-state Na-S pouch cell.

**Components**	**Size**	**Weight (g)**	**Required Quantity**
Na_xTiO_2/S cathode	7.5 cm × 8.5 cm	0.499	2 pieces
Na anode	7.5 cm × 8.5 cm × 100 μm	0.668	1 piece
PE	8.5 cm × 9.5 cm	0.352	2 pieces

The energy density (E_g) of the $Na_{0.25}TiO_2$-based pouch cells was calculated by accounting for all internal battery components, excluding the tabs and the aluminum-plastic packaging film, according to the following formula:

$$E_g = \frac{C \times V}{\sum M_i}$$

C: Capacity of pouch cells;

V: Average voltage of pouch cells;

M_i: Mass of cathode, anode and PE.

Table S4. Comparison of the electrochemical performance with previously reported Na-S pouch cells (C, A, and E stands for cathode, anode, and electrolyte, respectively.)

Cathode	Initial capacity at current density	Reversible capacity after cycle number	Energy density	(Based on the weight)	Ref.
**$Na_{0.25}TiO_2/S$**	**1158.8 mAh g^{-1} at 0.1C**	**684.1 mAh g^{-1} after 50 cycles**	**371.9 Wh kg^{-1}**	**(C+A+E)**	**This work**
S/PtNi-NPC	700 mAh g^{-1}, N/A	< 400 mAh g^{-1} after 60 cycles	240 Wh kg^{-1}	(C+A)	*Angew. Chem.* **2024**, 136, e202402274[13]
SPAN	807 mAh g^{-1} at 1C	~668 mAh g^{-1} after 23 cycles	N/A	(N/A)	*Adv. Funct. Mater.* **2022**, 32, 2201191[14]
S/P-Fe_2O_3@Fe-PPy	699 mAh g^{-1} at 0.03C	~300 mAh g^{-1} after 30 cycles	38 Wh kg^{-1}	(C+A+E)	*Angew. Chem.* **2023**, 135, e202217009[15]
C/S	750 mAh g^{-1} at 0.1C	661 mAh g^{-1} after 50 cycles	N/A	(N/A)	*Energy Environ. Sci.* **2024**, 17, 7416-7423[16]
SPAN	~600 mAh g^{-1} at 0.03C	~400 mAh g^{-1} after 50 cycles	N/A	(N/A)	*J. Mater. Chem. A* **2025**, 13, 6813-6825[17]
S	N/A, N/A	N/A	45.57 Wh kg^{-1}	(C+A+E)	*Carbon Energy* **2023**, 5(12), e371[18]
$FTe_{0.01}S_{0.99}PAN$	~639.5 mAh g^{-1} at 0.05C	~436 mAh g^{-1} after 50 cycles	312.5 Wh kg^{-1}	(C+A)	*Nano Energy* **2024**, 129, 110049[19]
SA Co-N/S	N/A, N/A	N/A	118.3 Wh kg^{-1}	(C+A+E)	*Nat. Commun.* **2025**, 16, 5827[20]

REFERENCES

(1) Shen, D. Y.; Zhao, G. L.; Jiang, T. L.; Liu, H. X.; Luo, R. H.; Zhang, Z. D.; Zhang, Z. W.; Chen, J. H.; Xu, J. W.; Feng, Y. C.; et al. A High-Capacity Manganese-Metal Battery with Dual-Storage Mechanism. *Angew. Chem. Int. Edit.* **2025**, *64* (15), e202423921. DOI: 10.1002/anie.202423921.

(2) Xu, F.; Yang, S. H.; Jiang, G. S.; Ye, Q.; Wei, B. Q.; Wang, H. Q. Fluorinated, Sulfur-Rich, Covalent Triazine Frameworks for Enhanced Confinement of Polysulfides in Lithium-Sulfur Batteries. *ACS Appl. Mater. Interfaces* **2017**, *9* (43), 37731–37738. DOI: 10.1021/acsami.7b10991.

(3) Wang, X.; He, L.; Xu, L. L.; Liu, Z. S.; Xiong, Y.; Zhou, W. Q.; Yao, H.; Wen, Y. P.; Geng, X.; Wu, R. M. Intelligent analysis of carbendazim in agricultural products based on a ZSHPC/MWCNT/SPE portable nanosensor combined with machine learning methods. *Anal. Methods* **2023**, *15* (5), 562–571. DOI: 10.1039/d2ay01779b.

(4) Nasu, A.; Otono, T.; Takayanagi, T.; Deguchi, M.; Sakuda, A.; Tatsumisago, M.; Hayashi, A. Utilizing reactive polysulfides flux Na_2S_x for the synthesis of sulfide solid electrolytes for all-solid-state sodium batteries. *Energy Storage Mater.* **2024**, *67*, 103307. DOI: 10.1016/J.Ensm.2024.103307.

(5) Reda, M.; Kupka, J.; Surace, Y.; Cupid, D. M.; Flandorfer, H. Unveiling the Thermal Behavior of SnS_2 Anodes Across Delithiation Stages. *Batteries* **2025**, *11* (10), 378. DOI: 10.3390/Batteries11100378.

(6) Zhou, D.; Chen, Y.; Li, B. H.; Fan, H. B.; Cheng, F. L.; Shanmukaraj, D.; Rojo, T.; Armand, M.; Wang, G. X. A Stable Quasi-Solid-State Sodium-Sulfur Battery. *Angew. Chem. Int. Edit.* **2018**, *57* (32), 10168–10172. DOI: 10.1002/anie.201805008.

(7) Cui, M. Y.; Yuan, S. S.; Jin, B.; Jiang, Q. Dual-functional in-situ gel polymer electrolyte for high-performance quasi-solid-state Na-S batteries. *J. Energy Chem.* **2025**, *109*, 241–250. DOI: 10.1016/j.jechem.2025.05.046.

(8) Huang, J. F.; Song, Z. G.; Wu, J. X.; Miao, Y. H.; Li, M. X.; Lin, D. J.; Zhu, K.; Chen, X. C.; Li, X. Y.; Chen, Y. M. Polymerized quasi-solid polymer electrolytes enabling void-free interfaces for room-temperature sodium-sulfur batteries. *Energy Mater. Devices* **2024**, *2* (4), 9370051. DOI: 10.26599/Emd.2024.9370051.

(9) Yu, X. W.; Manthiram, A. Sodium-Sulfur Batteries with a Polymer-Coated NASICON-type Sodium-Ion Solid Electrolyte. *Matter* **2019**, *1* (2), 439–451. DOI: 10.1016/j.matt.2019.03.008.

(10) Lim, D. H.; Agostini, M.; Ahn, J. H.; Matic, A. An Electrospun Nanofiber Membrane as Gel-Based Electrolyte for Room-Temperature Sodium-Sulfur Batteries. *Energy Technol.* **2018**, *6* (7), 1214–1219. DOI: 10.1002/ente.201800170.

(11) Hu, X. F.; Ni, Y. X.; Wang, C. A. L.; Wang, H.; Matios, E.; Chen, J.; Li, W. Y. Facile-Processed Nanocarbon-Promoted Sulfur Cathode for Highly Stable Sodium-Sulfur Batteries. *Cell Rep. Phys. Sci.* **2020**, *1* (2), 100015. DOI: 10.1016/J.Xcrp.2020.100015.

(12) Hu, X. F.; Matios, E.; Zhang, Y. W.; Wang, C. L.; Luo, J. M.; Li, W. Y. Enabling stable sodium metal cycling by sodiophilic interphase in a polymer electrolyte system. *J. Energy Chem.* **2021**, *63*, 305–311. DOI: 10.1016/j.jechem.2021.06.026.

(13) Zhang, H.; Wang, M. L.; Song, B.; Huang, X. L.; Zhang, W. L.; Zhang, E. R.; Cheng, Y. W.; Lu, K. Quasi-Solid Sulfur Conversion for Energetic All-Solid-State Na-S Battery. *Angew. Chem. Int. Edit.* **2024**, *63* (19), e202402274. DOI: 10.1002/anie.202402274.

(14) Murugan, S.; Klostermann, S. V.; Schützendübe, P.; Richter, G.; Kästner, J.; Buchmeiser, M. R. Stable Cycling of Room-Temperature Sodium-Sulfur Batteries Based on an In Situ Crosslinked Gel Polymer Electrolyte. *Adv. Funct. Mater.* **2022**, *32* (32), 2201191. DOI: 10.1002/Adfm.202201191.

(15) Zhang, H.; Song, B.; Zhang, W. W.; An, B. W.; Fu, L.; Lu, S. T.; Cheng, Y. W.; Chen, Q. W.; Lu, K. Bidirectional Tandem Electrocatalysis Manipulated Sulfur Speciation Pathway for High-Capacity and Stable Na-S Battery. *Angew. Chem. Int. Edit.* **2023**, *62* (6), e202217009. DOI: 10.1002/anie.202217009.

(16) He, J. R.; Bhargav, A.; Manthiram, A. Long-life sodium-sulfur batteries enabled by super-sodiophilic seeds. *Energy Environ. Sci.* **2024**, *17* (19), 7416–7423. DOI: 10.1039/d4ee02996h.

(17) Soni, C. B.; Barik, S. K.; Vineeth, S. K.; Yadav, B.; Chandra, M.; Sungjemmenla; Sanjaykumar, C.; Kumar, H.; Kumar, V. Sodium metal anodes with multiphasic interphases for room temperature sodium-sulfur pouch cells. *J. Mater. Chem. A* **2025**, *13* (9), 6813–6825. DOI: 10.1039/d4ta08118h.

(18) Yang, A. K.; Ye, R. J.; Song, H. M.; Lu, Q. Q.; Wang, X. C.; Dashjav, E.; Yao, K.; Grüner, D.; Ma, Q. L.; Tietz, F.; et al. Pressureless all-solid-state Na/S batteries with self-supporting $Na_5YSi_4O_{12}$ scaffolds. *Carbon Energy* **2023**, *5* (12), e371. DOI: 10.1002/cey2.371.

(19) Wu, Q.; Zhang, W.; Qin, M. S.; Zhong, W.; Yan, H.; Zhu, H. L.; Cheng, S. J.; Xie, J. Tellurium doped sulfurized polyacrylonitrile nanoflower for high-energy-density, long-lifespan sodium-sulfur batteries. *Nano Energy* **2024**, *129*, 110049. DOI: 10.1016/J.Nanoen.2024.110049.

(20) Bai, R. L.; Yao, Y.; Lin, Q. S.; Wu, L. Z.; Li, Z.; Wang, H. J.; Ma, M. Z.; Mu, D.; Hu, L. X.; Yang, H.; et al. Preferable single-atom catalysts enabled by natural language processing for high energy density Na-S batteries. *Nat. Commun.* **2025**, *16* (1), 5827. DOI: 10.1038/S41467-025-60931-X.
